# Sensory and decision-making processes underlying perceptual adaptation

**DOI:** 10.1167/18.8.10

**Published:** 2018-08-22

**Authors:** Nathan Witthoft, Long Sha, Jonathan Winawer, Roozbeh Kiani

**Affiliations:** jonathan.winawer@nyu.edu; roozbeh@nyu.edu; Department of Psychology, New York University, New York, NY, USA; Department of Psychology, Stanford University, Stanford, CA, USA; Center for Neural Science, New York University, New York, NY, USA; Department of Psychology and the Center for Neural Science, New York University, New York, NY, USA; Department of Psychology and the Center for Neural Science, New York University, New York, NY, USA; Neuroscience Institute, NYU Langone Medical Center, New York, NY, USA

**Keywords:** adaptation, sensitivity, decision bound, reaction time, facial expression

## Abstract

Perceptual systems adapt to their inputs. As a result, prolonged exposure to particular stimuli alters judgments about subsequent stimuli. This phenomenon is commonly assumed to be sensory in origin. Changes in the decision-making process, however, may also be a component of adaptation. Here, we quantify sensory and decision-making contributions to adaptation in a facial expression paradigm. As expected, exposure to happy or sad expressions shifts the psychometric function toward the adaptor. More surprisingly, response times show both an overall decline and an asymmetry, with faster responses opposite the adapting category, implicating a substantial change in the decision-making process. Specifically, we infer that sensory changes from adaptation are accompanied by changes in how much sensory information is accumulated for the two choices. We speculate that adaptation influences implicit expectations about the stimuli one will encounter, causing modifications in the decision-making process as part of a normative response to a change in context.

## Introduction

Perceptual adaptation has long been an important tool for studying visual representation. The premise behind many adaptation studies is that if judgments about a stimulus dimension are systematically influenced by adaptation, then that dimension is part of the sensory code. Hence, adaptation has been called the “psychophysicist's electrode” (Frisby, 1980), or put another way, “if it adapts, it's there” (Mollon, 1974). This logic has been used to infer properties of the neural code from behavior, including color opponency (Hering, 1874), spatial frequency channels (Blakemore & Campbell, 1969; Campbell & Robson, 1968), and directional motion selectivity (Exner, 1887). When using adaptation to study sensory representations, changes in the observer's decision-making strategy typically do not play an important role: Despite some recent interest in the decision-level normalization in adaptation (Mather & Sharman, 2015), decisional effects are typically not assessed in adaptation studies, or are considered an unwanted artifact best eliminated by experimental design (e.g., Morgan, 2013, 2014).

Here we take a different approach. Given that the nervous system is constantly adjusting to the local environment, adaptation is likely an integral part of its ordinary operation, and is therefore important to understand in and of itself. Adaptation presumably confers an advantage to an observer by increasing the likelihood of responding appropriately given the observed or expected stimuli in the current environment (Rieke & Rudd, 2009; Webster, 2015). If part of this process is a change in decision strategy (in addition to changes in sensory representation), then the manner in which decisions are made needs to be accounted for in any complete model of adaptation. In this paper, we simultaneously investigated decisional and representational effects arising from perceptual adaptation, and sought to quantify the contribution of both types of effects on judgments made during adaptation.

To accomplish this, we used a common experimental design for probing adaptation, in which an observer views a prolonged or repeated stimulus while making interleaved judgments about test stimuli. The particular stimulus domain we used was facial expression, a natural stimulus category that has been shown to produce strong and reliable effects of adaptation (Fox & Barton, 2007; Hsu & Young, 2004; Russell & Fehr, 1987; Webster, Kaping, Mizokami, & Duhamel, 2004; Xu, Dayan, Lipkin, & Qian, 2008); for example, a facial expression judged as neutral when the observer is unadapted will be reported as sad following prolonged viewing of a happy facial expression. The difference between the pre- and post-adaptation judgments of the same stimulus could arise because of a representational change or a change in the criterion for specifying one or the other choice, or a combination of these. We sought to account for these effects within a single experimental paradigm, in which we recorded both choice and response time. The two dependent measures can be used in tandem to constrain a dynamic model of the decision-making process (Bogacz, 2007; Brunton, Botvinick, & Brody, 2013; Donkin, Brown, Heathcote, & Wagenmakers, 2011; Purcell & Kiani, 2016b; Shadlen & Kiani, 2013; Smith & Ratcliff, 2004; Usher & McClelland, 2001). One general form of a decision model, an accumulation to bound model, has been used to link the psychometric function (choice as a function of stimulus strength) and the chronometric function (response time as a function of stimulus strength) (Link, 1992; Ratcliff & McKoon, 2008). By simultaneously accounting for both types of data, the model can be used as a tool to understand how decisions change under conditions of adaptation.

Importantly, effects at different stages of the perceptual decision-making process—from the accumulation of evidence to arriving at a stop criterion—translate to different patterns of choice and response times. These patterns are made transparent in the framework of an accumulation to bound model of the decision-making process. The model assumes that a stimulus gives rise to sensory evidence that fluctuates over time due to stochasticity of neural responses, and that the observer accumulates this noisy evidence until a decision boundary is reached. Models of this general form have been applied to a wide range of perceptual tasks, accounting for both behavioral and neural data (Gold & Shadlen, 2007; Purcell et al., 2010; Ratcliff, Cherian, & Segraves, 2003), including face perception (Okazawa, Sha, & Kiani, 2016). In the framework of this model, a pure representational effect modifies the sensory evidence furnished by the test stimulus and is expected to cause equivalent shifts in the subject's psychometric and chronometric functions. In contrast, a change in the starting point or termination criterion of the evidence accumulation process can introduce asymmetries in the chronometric function; for example, if as a result of adaptation, the termination criterion becomes smaller for one of two choices, then we would expect faster responses when this choice is made compared to the alternative, even for an identical stimulus. If, on the other hand, the termination criterion changes symmetrically for the two decisions, say both decision bounds becoming smaller, responses would be more susceptible to noise in the evidence accumulation process. As a result, the observer will be expected to make less consistent judgments (a shallower psychometric function), and to exhibit shorter response times for both decisions.

By examining the pattern of response times and choice during facial expression adaptation paradigms, we were able to address several questions. First, we asked whether the change in behavior due to adaptation is well described within the framework of a drift-diffusion model—specifically, is a change in model parameters sufficient to capture the main patterns by which the psychometric and chronometric functions change under conditions of adaptation? Second, in Experiment 1, using a method of constant stimuli, we quantified the degree to which representational and decisional factors were altered by adaptation, and we weighed the relative contributions of these two factors to the shift in the psychometric function. Third, in Experiment 2, we asked whether and how these patterns change when the range of test stimuli is chosen to ensure that the participant makes an approximately equal proportion of responses under all conditions. Together, the results from the two experiments show that under ordinary experimental methods for probing adaptation, participants tend to make substantial changes to their decision strategy, and that the decision-making and representational changes combine to produce the shifts in judgments typically associated with adaptation.

## Methods

### Participants

Six volunteers (four male, 18–45 years old) participated in the experiment, either at New York University (four participants) or Stanford University (two participants). All participants had normal visual acuity or corrected-to-normal vision with glasses or contact lenses. The experimental protocol was approved by the New York University Committee on Activities Involving Human Subjects and by the Stanford University Institutional Review Board. Informed written consents were obtained from all participants prior to the study. Two of the participants were authors (NW, LS). The other four were naive. Each participant completed 5,830–7,480 trials (median: 6,655) over approximately 13 one-hr sessions.

### Stimuli

**Figure 1:**
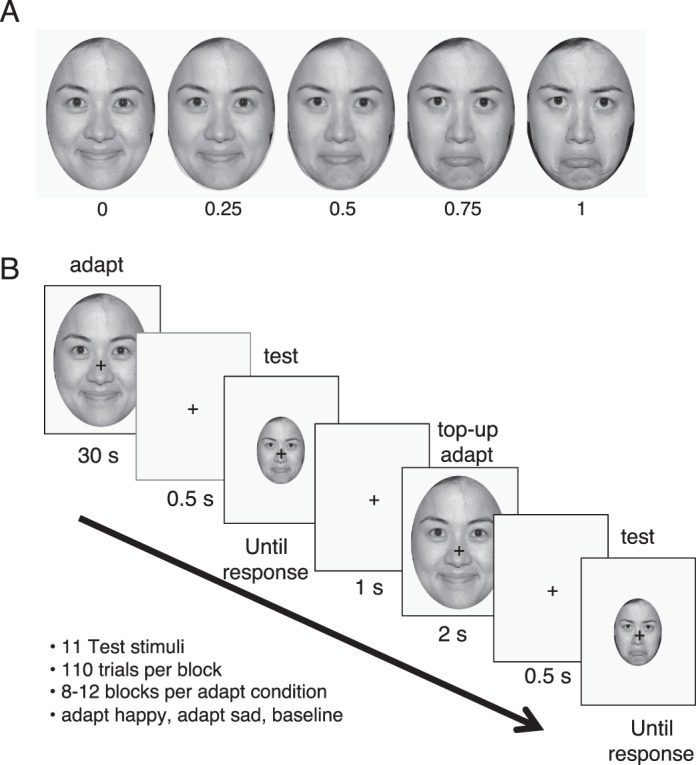
Stimulus set and procedure for Experiment 1. (A) Stimulus morph line from happy to sad. The end points, stim-0 and stim-1 were the two adapting stimuli, and defined the range from happy to sad. The intermediate stimuli smoothly transitioned between the two emotional expressions. (B) Trial structure. The participant viewed the adapting stimulus for 30 s at the start of each block, fixating the black cross. After a 0.5-s delay, the test stimulus was presented, scaled down by 50%, and remained on the screen until the participant made a key press to indicate a sad or happy facial expression. Each block consisted of 110 trials, with each trial testing one of 11 stimuli that spanned the morph line. After the first trial, the participant viewed the same adapting stimulus, but for 2 s rather than 30 s. The prototype stimuli were adopted from photographs of Model #18 in the MacBrain Face Stimulus Set (https://www.macbrain.org/resources.htm; the model gave permission to reprint the images in scientific journals).

We constructed a stimulus set of 41 grayscale images of faces, windowed within an oval aperture to mask the ears and hairline (Figure 1A). The stimulus set depicted a single person with emotional facial expressions spanning a range from happy to sad. We adopt the arbitrary convention of defining a stimulus axis that increases for sad expressions, from 0 (happy) to 1 (sad). We derived the images from photographs in the MacBrain Face Stimulus Set developed by Nim Tottenham and made publicly available to the scientific community (http://www.macbrain.org/resources.htm). The database contains color photographs of actors instructed to make various facial expressions—fearful, happy, sad, angry, surprised, calm, neutral, disgusted—with the mouth either open or closed (16 photographs per model). Our stimuli were made from two of these, the happy and sad closed-mouth photographs of Model #18. The expressions in the database were validated by independent test subjects, with 91% and 95% accuracy on an eight-choice decision for our two stimuli (supplementary table 2A in Tottenham et al., 2009). A subset of the models, including #18, gave permission to reprint the images in scientific journals (https://www.macbrain.org/resources.htm).

We converted the happy and sad images to grayscale and used these as the endpoints of our continuum of emotional expression. We made 39 intermediate stimuli using morphing software (Morph 2.1, Norrkross software, available at http://www.norrkross.com/software/morphx/morphx.php). The 41 stimuli were equally spaced on the morph trajectory. To minimize artifacts in the intermediate stimuli such as the transparent overlay of noncorresponding regions from the two endpoints, we hand-labeled about 50 pairs of corresponding points in the two end-point images. The corresponding points were placed along the outer border of the face and on and around internal features (eyes, nose, mouth, brow). These points guide the geometric warping, which helps ensure that cross-fading only occurs between corresponding image regions. After morphing, all stimuli were windowed within an oval aperture with a 1.35 aspect ratio (height to width).

Visual inspection by the authors confirmed that the morphed stimuli appeared to have facial expressions that varied smoothly from happy to sad and looked as natural (artifact-free) as the two endpoints. As we report in the Results, the regularity in perceived stimulus strength along the happy-to-sad continuum was also assessed quantitatively using psychophysical methods. As there were 41 stimuli in our set—original happy and sad endpoints and the 39 intermediate stimuli—we defined the stimulus strength by dividing the image number (0 to 40) by 40. The complete set of the stimuli in our morph trajectory is provided as supplementary data (Supplementary Figure S1).

During the experiment, participants sat in front of a flat-screen CRT display and, using a chin rest, maintained a viewing distance of 52–57 cm. The stimuli were presented at either 13.5 × 10 degrees of visual angle (adapting stimuli) or 6.25 × 5 degrees of visual angle (test stimuli).

### Procedure

#### Summary

Each observer participated in two experiments to measure adaptation to emotional expression, completing 27–40 blocks of trials (median: 30) per experiment, with 110 trials per block. Each 1-hr session comprised multiple blocks and each block lasted about 6–8 min (depending on the participant's response time), with the opportunity to rest between blocks.

#### Experiment 1: Fixed stimulus range

During each block of trials, participants adapted to one of the two endpoint images in our stimulus set—the happy face (stimulus 0) or sad face (stimulus 1)—or to no stimulus (blank screen with a fixation cross), and made happy/sad judgments about 11 intermediate stimuli (levels 0.125, 0.375, 0.400, 0.425, 0.450, 0.475, 0.500, 0.525, 0.550, 0.575, and 0.875). The 11 test stimuli appeared 10 times in random order. In each session, participants completed four to eight blocks of trials—typically two consecutive blocks of no-adaptation and four consecutive blocks of either adapt-happy or adapt-sad—so that across the multiple sessions there was an equal number of each of the three types of adaptation blocks. We considered the first six blocks for each adaptation condition to be training blocks, and did not use them for subsequent analysis.

Every block began with a long adaptation trial, in which the participant viewed the adapt-happy stimulus, adapt-sad stimulus, or no image (a uniform gray screen) continuously for 30 s while maintaining fixation on a small black cross in the center of the screen (Figure 1B). After the 30 s of adaptation, the participant viewed a brief blank screen with a fixation cross (0.5-s interstimulus interval) followed by the test stimulus. The test stimulus was half the size of the adapting stimulus in length and width. The purpose of the size change was to decorrelate the local image features between the adapting and test stimuli. The transfer of adaptation across a large change in image size supports the interpretation that the aftereffect is linked to global properties of the image rather than to the specific local luminance and contrast; even very small shifts in position (0.2°) between adaptor and test have been shown to reduce or eliminate the influence of adaptation from nonface specific mechanisms (Xu et al., 2008). The test stimulus remained on the screen until the participant pressed a key to indicate their response (x for happy, m for sad). Following the button press, there was a 1-s intertrial interval with a blank screen. For the subsequent 109 trials in the block, the trial structure was the same except that the adapting stimulus (or blank screen) was viewed for only 2 s instead of 30 s. This method of using a long initial adaptation period followed by shorter periods of top-up adaptation before each test stimulus is typical of adaptation experiments (Clifford & Rhodes, 2005). The adapting stimulus was the same throughout the block of 110 trials. Participants were not provided feedback. They were only asked to provide consistent responses across blocks.

#### Experiment 2: Balanced responses

**Figure 2:**
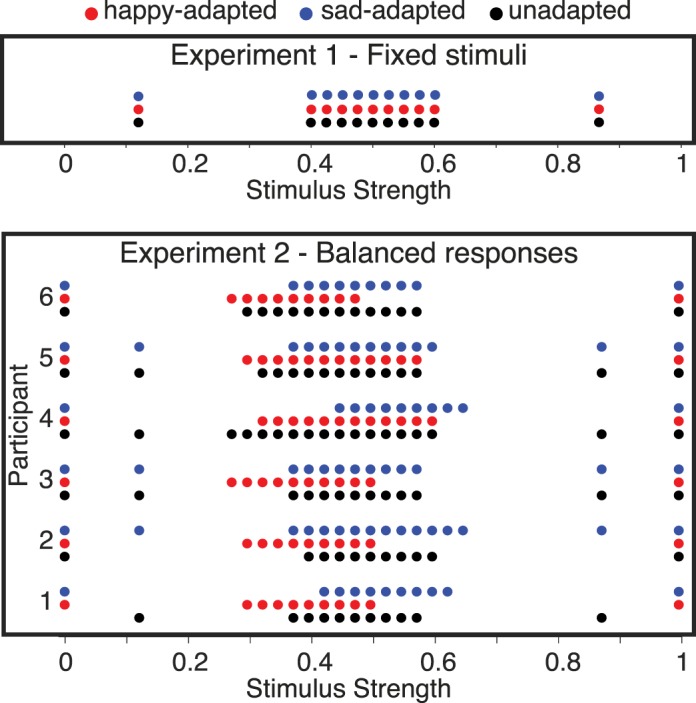
Test stimuli for Experiments 1 and 2. The upper panel shows the test stimuli for Experiment 1 (fixed stimulus set). Each row shows one adaptation condition indicated by the dot colors (blue: adapt sad; red: adapt happy; black: unadapted). The test stimuli were identical for all participants and all adapting conditions. The test comprised nine equally spaced stimuli near the midpoint of our stimulus set, and two stimuli near the extremes. The middle stimuli are most useful for estimating the steep portion of the psychometric curves, and the more extreme stimuli for estimating the asymptotes. The lower panel shows the stimuli for Experiment 2 (balanced responses). These stimuli differed for each participant and each adapting condition. The stimuli were chosen in order to achieve an approximately equal probability of happy and sad responses for each participant and each adapting condition, as determined in practice blocks. To obtain balanced responses, the range of test stimuli was generally shifted towards the adapting stimulus.

Experiment 2 was identical to Experiment 1 except for the set of test stimuli. In Experiment 1, the set of test stimuli was the same across observers and adapting conditions (Figure 2, top). When adapting to the happy face, most responses were “sad” (58.3% pooled across observers), and when adapting to the sad face, most responses were “happy” (62.8% pooled across observers; see Table 1). In Experiment 2, we altered the range of stimuli per observer and per adapting condition in order to balance the proportion of sad and happy responses. This was achieved by fitting a psychometric function to the judgments made in each practice block, and recentering the middle stimuli to the point of subjective equality (PSE) from the psychometric fit for the subsequent blocks. In the first session of each adaptation condition, we estimated the PSE over multiple blocks and repeated the measurements until a stable PSE was observed across consecutive blocks. In subsequent sessions, the first block was used to ensure stability of PSE across days. In the case that the PSE changed significantly, we reestimated the new PSE over multiple blocks and used the new value for recentering the stimuli; as a result, some participants had greater than 11 stimulus levels. This procedure was applied independently for each adaptation condition. The calibration was successful, in that the mean responses were close to 50% for each observer in each condition for Experiment 2 (Table 1).

**Table 1:**
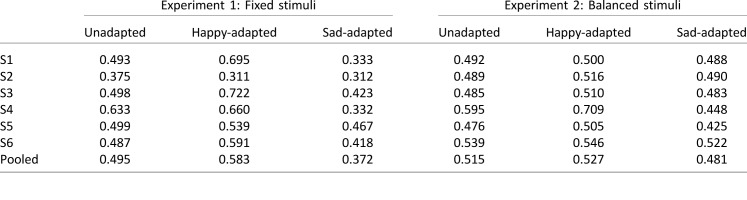
Probability of sad responses in Experiments 1 and 2.

### Data analysis

The broad goal of our analysis was to test whether (and how) a computational model of decision making could account for the distribution of response times and choices for each test stimulus strength in each adaptation condition. Accurately modeling these distributions requires a large number of trials per condition. Even with about 6,000 trials per participant, there was limited power to fit models to individual participant's data (∼1,000 trials for each adaptation condition in each experiment). For the primary analysis, we therefore combined data across participants. We also show individual participant's data and model fits in the supplementary material (Supplementary Figure S4), and for each analysis in the main text we report the number of participants showing statistically significant results consistent with the group data. Finally, we devised a bootstrap procedure to aggregate individual participant's results across the tested population. In each iteration of the bootstrap procedure, we calculated the average of the quantity of interest (e.g., change of decision bound) for each subject by sampling and averaged the results across subjects. We repeated the process 10^4^ times to make a bootstrap distribution of the across-subject averages. The *p* values were based on this distribution (e.g., proportion of average bound changes that exceeded zero).

#### Combining data across participants

For both experiments, trials with response times greater than 5 s (0.06% of trials for Experiment 1 and 0.03% for Experiment 2) were considered outliers and were omitted from analysis.

For Experiment 1, in which each participant viewed the same set of test stimuli, combining data was straightforward: We concatenated the trials across participants for each of the 33 combinations of adaptation conditions (happy, sad, and neutral) and test items (11 stimulus strengths), and then analyzed the group data with three models, one per adaptation condition.

For Experiment 2, the set of test stimuli varied across adaptation condition and participant, both in terms of the number of stimuli tested and the range of the stimuli. To combine across participants, the stimuli were aligned and binned in the following manner. First, we computed the PSE separately for each participant in each adaptation condition by fitting a logistic regression to the judgments (see the Effect of adaptation on choice section below). This value was, by design, close to the middle test stimulus for each participant and adaptation condition, since the test stimuli were chosen based on performance during practice blocks with the explicit goal of centering the range on the individual's PSE. For each of the three adaptation conditions (happy-adapted, sad-adapted, and unadapted), we averaged the PSEs across participants, yielding a condition-specific PSE. We then added to the stimulus strength the difference between the group average PSE and the individual's PSE, thereby aligning the stimulus strengths used across participants to a common reference for each adaptation condition. After alignment, we binned the data points into 19 discrete bins, and dropped any bin that fewer than two-thirds of participants contributed to, leaving 11 bins per adaptation condition. Once data were combined across participants, model fitting was identical for the two experiments.

The identical binning procedure was also used for individual participant analysis, except that the data were not combined across participants.

#### Effect of adaptation on choice

To calculate the PSE for each adaptation condition, we used the following logistic regression:

\newcommand{\bialpha}{\boldsymbol{\alpha}}\newcommand{\bibeta}{\boldsymbol{\beta}}\newcommand{\bigamma}{\boldsymbol{\gamma}}\newcommand{\bidelta}{\boldsymbol{\delta}}\newcommand{\bivarepsilon}{\boldsymbol{\varepsilon}}\newcommand{\bizeta}{\boldsymbol{\zeta}}\newcommand{\bieta}{\boldsymbol{\eta}}\newcommand{\bitheta}{\boldsymbol{\theta}}\newcommand{\biiota}{\boldsymbol{\iota}}\newcommand{\bikappa}{\boldsymbol{\kappa}}\newcommand{\bilambda}{\boldsymbol{\lambda}}\newcommand{\bimu}{\boldsymbol{\mu}}\newcommand{\binu}{\boldsymbol{\nu}}\newcommand{\bixi}{\boldsymbol{\xi}}\newcommand{\biomicron}{\boldsymbol{\micron}}\newcommand{\bipi}{\boldsymbol{\pi}}\newcommand{\birho}{\boldsymbol{\rho}}\newcommand{\bisigma}{\boldsymbol{\sigma}}\newcommand{\bitau}{\boldsymbol{\tau}}\newcommand{\biupsilon}{\boldsymbol{\upsilon}}\newcommand{\biphi}{\boldsymbol{\phi}}\newcommand{\bichi}{\boldsymbol{\chi}}\newcommand{\bipsi}{\boldsymbol{\psi}}\newcommand{\biomega}{\boldsymbol{\omega}}\begin{equation}\tag{1}logit\left[ {P\left( {sad} \right)} \right] = {\beta _0}\left( {S + {\beta _1}} \right)\end{equation}

where $\newcommand{\bialpha}{\boldsymbol{\alpha}}\newcommand{\bibeta}{\boldsymbol{\beta}}\newcommand{\bigamma}{\boldsymbol{\gamma}}\newcommand{\bidelta}{\boldsymbol{\delta}}\newcommand{\bivarepsilon}{\boldsymbol{\varepsilon}}\newcommand{\bizeta}{\boldsymbol{\zeta}}\newcommand{\bieta}{\boldsymbol{\eta}}\newcommand{\bitheta}{\boldsymbol{\theta}}\newcommand{\biiota}{\boldsymbol{\iota}}\newcommand{\bikappa}{\boldsymbol{\kappa}}\newcommand{\bilambda}{\boldsymbol{\lambda}}\newcommand{\bimu}{\boldsymbol{\mu}}\newcommand{\binu}{\boldsymbol{\nu}}\newcommand{\bixi}{\boldsymbol{\xi}}\newcommand{\biomicron}{\boldsymbol{\micron}}\newcommand{\bipi}{\boldsymbol{\pi}}\newcommand{\birho}{\boldsymbol{\rho}}\newcommand{\bisigma}{\boldsymbol{\sigma}}\newcommand{\bitau}{\boldsymbol{\tau}}\newcommand{\biupsilon}{\boldsymbol{\upsilon}}\newcommand{\biphi}{\boldsymbol{\phi}}\newcommand{\bichi}{\boldsymbol{\chi}}\newcommand{\bipsi}{\boldsymbol{\psi}}\newcommand{\biomega}{\boldsymbol{\omega}}logit\left( p \right) = log\left( {{p \over {1 - p}}} \right)$, $\newcommand{\bialpha}{\boldsymbol{\alpha}}\newcommand{\bibeta}{\boldsymbol{\beta}}\newcommand{\bigamma}{\boldsymbol{\gamma}}\newcommand{\bidelta}{\boldsymbol{\delta}}\newcommand{\bivarepsilon}{\boldsymbol{\varepsilon}}\newcommand{\bizeta}{\boldsymbol{\zeta}}\newcommand{\bieta}{\boldsymbol{\eta}}\newcommand{\bitheta}{\boldsymbol{\theta}}\newcommand{\biiota}{\boldsymbol{\iota}}\newcommand{\bikappa}{\boldsymbol{\kappa}}\newcommand{\bilambda}{\boldsymbol{\lambda}}\newcommand{\bimu}{\boldsymbol{\mu}}\newcommand{\binu}{\boldsymbol{\nu}}\newcommand{\bixi}{\boldsymbol{\xi}}\newcommand{\biomicron}{\boldsymbol{\micron}}\newcommand{\bipi}{\boldsymbol{\pi}}\newcommand{\birho}{\boldsymbol{\rho}}\newcommand{\bisigma}{\boldsymbol{\sigma}}\newcommand{\bitau}{\boldsymbol{\tau}}\newcommand{\biupsilon}{\boldsymbol{\upsilon}}\newcommand{\biphi}{\boldsymbol{\phi}}\newcommand{\bichi}{\boldsymbol{\chi}}\newcommand{\bipsi}{\boldsymbol{\psi}}\newcommand{\biomega}{\boldsymbol{\omega}}P\left( {sad} \right)$ is the probability of choosing “sad,” and *S* is the stimulus strength, expressed as a number between 0 (happy) and 1 (sad). $\newcommand{\bialpha}{\boldsymbol{\alpha}}\newcommand{\bibeta}{\boldsymbol{\beta}}\newcommand{\bigamma}{\boldsymbol{\gamma}}\newcommand{\bidelta}{\boldsymbol{\delta}}\newcommand{\bivarepsilon}{\boldsymbol{\varepsilon}}\newcommand{\bizeta}{\boldsymbol{\zeta}}\newcommand{\bieta}{\boldsymbol{\eta}}\newcommand{\bitheta}{\boldsymbol{\theta}}\newcommand{\biiota}{\boldsymbol{\iota}}\newcommand{\bikappa}{\boldsymbol{\kappa}}\newcommand{\bilambda}{\boldsymbol{\lambda}}\newcommand{\bimu}{\boldsymbol{\mu}}\newcommand{\binu}{\boldsymbol{\nu}}\newcommand{\bixi}{\boldsymbol{\xi}}\newcommand{\biomicron}{\boldsymbol{\micron}}\newcommand{\bipi}{\boldsymbol{\pi}}\newcommand{\birho}{\boldsymbol{\rho}}\newcommand{\bisigma}{\boldsymbol{\sigma}}\newcommand{\bitau}{\boldsymbol{\tau}}\newcommand{\biupsilon}{\boldsymbol{\upsilon}}\newcommand{\biphi}{\boldsymbol{\phi}}\newcommand{\bichi}{\boldsymbol{\chi}}\newcommand{\bipsi}{\boldsymbol{\psi}}\newcommand{\biomega}{\boldsymbol{\omega}}{\beta _0}$ and $\newcommand{\bialpha}{\boldsymbol{\alpha}}\newcommand{\bibeta}{\boldsymbol{\beta}}\newcommand{\bigamma}{\boldsymbol{\gamma}}\newcommand{\bidelta}{\boldsymbol{\delta}}\newcommand{\bivarepsilon}{\boldsymbol{\varepsilon}}\newcommand{\bizeta}{\boldsymbol{\zeta}}\newcommand{\bieta}{\boldsymbol{\eta}}\newcommand{\bitheta}{\boldsymbol{\theta}}\newcommand{\biiota}{\boldsymbol{\iota}}\newcommand{\bikappa}{\boldsymbol{\kappa}}\newcommand{\bilambda}{\boldsymbol{\lambda}}\newcommand{\bimu}{\boldsymbol{\mu}}\newcommand{\binu}{\boldsymbol{\nu}}\newcommand{\bixi}{\boldsymbol{\xi}}\newcommand{\biomicron}{\boldsymbol{\micron}}\newcommand{\bipi}{\boldsymbol{\pi}}\newcommand{\birho}{\boldsymbol{\rho}}\newcommand{\bisigma}{\boldsymbol{\sigma}}\newcommand{\bitau}{\boldsymbol{\tau}}\newcommand{\biupsilon}{\boldsymbol{\upsilon}}\newcommand{\biphi}{\boldsymbol{\phi}}\newcommand{\bichi}{\boldsymbol{\chi}}\newcommand{\bipsi}{\boldsymbol{\psi}}\newcommand{\biomega}{\boldsymbol{\omega}}{\beta _1}$ are regression coefficients that account for the slope and bias of the psychometric function. The PSE was defined as the stimulus strength for which the probability of the two choices became equal (0.5), that is $\newcommand{\bialpha}{\boldsymbol{\alpha}}\newcommand{\bibeta}{\boldsymbol{\beta}}\newcommand{\bigamma}{\boldsymbol{\gamma}}\newcommand{\bidelta}{\boldsymbol{\delta}}\newcommand{\bivarepsilon}{\boldsymbol{\varepsilon}}\newcommand{\bizeta}{\boldsymbol{\zeta}}\newcommand{\bieta}{\boldsymbol{\eta}}\newcommand{\bitheta}{\boldsymbol{\theta}}\newcommand{\biiota}{\boldsymbol{\iota}}\newcommand{\bikappa}{\boldsymbol{\kappa}}\newcommand{\bilambda}{\boldsymbol{\lambda}}\newcommand{\bimu}{\boldsymbol{\mu}}\newcommand{\binu}{\boldsymbol{\nu}}\newcommand{\bixi}{\boldsymbol{\xi}}\newcommand{\biomicron}{\boldsymbol{\micron}}\newcommand{\bipi}{\boldsymbol{\pi}}\newcommand{\birho}{\boldsymbol{\rho}}\newcommand{\bisigma}{\boldsymbol{\sigma}}\newcommand{\bitau}{\boldsymbol{\tau}}\newcommand{\biupsilon}{\boldsymbol{\upsilon}}\newcommand{\biphi}{\boldsymbol{\phi}}\newcommand{\bichi}{\boldsymbol{\chi}}\newcommand{\bipsi}{\boldsymbol{\psi}}\newcommand{\biomega}{\boldsymbol{\omega}}PSE = - {\beta _1}$. Regression coefficients in Equation 1 and subsequent logistic regressions were calculated using maximum likelihood fitting. Regression parameters and standard errors of Equation 1 for each participant and for the pooled data across participants are in Supplementary Table S1. We quantified the shift of psychometric functions as the difference of the PSE of adapted and unadapted conditions. Standard error of the shift was calculated by bootstrapping.

To test for changes in the slope of the psychometric function in adapted and unadapted conditions, we used the following logistic regression:

\newcommand{\bialpha}{\boldsymbol{\alpha}}\newcommand{\bibeta}{\boldsymbol{\beta}}\newcommand{\bigamma}{\boldsymbol{\gamma}}\newcommand{\bidelta}{\boldsymbol{\delta}}\newcommand{\bivarepsilon}{\boldsymbol{\varepsilon}}\newcommand{\bizeta}{\boldsymbol{\zeta}}\newcommand{\bieta}{\boldsymbol{\eta}}\newcommand{\bitheta}{\boldsymbol{\theta}}\newcommand{\biiota}{\boldsymbol{\iota}}\newcommand{\bikappa}{\boldsymbol{\kappa}}\newcommand{\bilambda}{\boldsymbol{\lambda}}\newcommand{\bimu}{\boldsymbol{\mu}}\newcommand{\binu}{\boldsymbol{\nu}}\newcommand{\bixi}{\boldsymbol{\xi}}\newcommand{\biomicron}{\boldsymbol{\micron}}\newcommand{\bipi}{\boldsymbol{\pi}}\newcommand{\birho}{\boldsymbol{\rho}}\newcommand{\bisigma}{\boldsymbol{\sigma}}\newcommand{\bitau}{\boldsymbol{\tau}}\newcommand{\biupsilon}{\boldsymbol{\upsilon}}\newcommand{\biphi}{\boldsymbol{\phi}}\newcommand{\bichi}{\boldsymbol{\chi}}\newcommand{\bipsi}{\boldsymbol{\psi}}\newcommand{\biomega}{\boldsymbol{\omega}}\begin{equation}\tag{2}logit\left[ {P\left( {sad} \right)} \right] = \left( {{\beta _0} + {\beta _4}{L_3}} \right)\left( {S + {\beta _1} + {\beta _2}{L_1} + {\beta _3}{L_2}} \right)\end{equation}

where $\newcommand{\bialpha}{\boldsymbol{\alpha}}\newcommand{\bibeta}{\boldsymbol{\beta}}\newcommand{\bigamma}{\boldsymbol{\gamma}}\newcommand{\bidelta}{\boldsymbol{\delta}}\newcommand{\bivarepsilon}{\boldsymbol{\varepsilon}}\newcommand{\bizeta}{\boldsymbol{\zeta}}\newcommand{\bieta}{\boldsymbol{\eta}}\newcommand{\bitheta}{\boldsymbol{\theta}}\newcommand{\biiota}{\boldsymbol{\iota}}\newcommand{\bikappa}{\boldsymbol{\kappa}}\newcommand{\bilambda}{\boldsymbol{\lambda}}\newcommand{\bimu}{\boldsymbol{\mu}}\newcommand{\binu}{\boldsymbol{\nu}}\newcommand{\bixi}{\boldsymbol{\xi}}\newcommand{\biomicron}{\boldsymbol{\micron}}\newcommand{\bipi}{\boldsymbol{\pi}}\newcommand{\birho}{\boldsymbol{\rho}}\newcommand{\bisigma}{\boldsymbol{\sigma}}\newcommand{\bitau}{\boldsymbol{\tau}}\newcommand{\biupsilon}{\boldsymbol{\upsilon}}\newcommand{\biphi}{\boldsymbol{\phi}}\newcommand{\bichi}{\boldsymbol{\chi}}\newcommand{\bipsi}{\boldsymbol{\psi}}\newcommand{\biomega}{\boldsymbol{\omega}}{L_i}$ are indicator variables: $\newcommand{\bialpha}{\boldsymbol{\alpha}}\newcommand{\bibeta}{\boldsymbol{\beta}}\newcommand{\bigamma}{\boldsymbol{\gamma}}\newcommand{\bidelta}{\boldsymbol{\delta}}\newcommand{\bivarepsilon}{\boldsymbol{\varepsilon}}\newcommand{\bizeta}{\boldsymbol{\zeta}}\newcommand{\bieta}{\boldsymbol{\eta}}\newcommand{\bitheta}{\boldsymbol{\theta}}\newcommand{\biiota}{\boldsymbol{\iota}}\newcommand{\bikappa}{\boldsymbol{\kappa}}\newcommand{\bilambda}{\boldsymbol{\lambda}}\newcommand{\bimu}{\boldsymbol{\mu}}\newcommand{\binu}{\boldsymbol{\nu}}\newcommand{\bixi}{\boldsymbol{\xi}}\newcommand{\biomicron}{\boldsymbol{\micron}}\newcommand{\bipi}{\boldsymbol{\pi}}\newcommand{\birho}{\boldsymbol{\rho}}\newcommand{\bisigma}{\boldsymbol{\sigma}}\newcommand{\bitau}{\boldsymbol{\tau}}\newcommand{\biupsilon}{\boldsymbol{\upsilon}}\newcommand{\biphi}{\boldsymbol{\phi}}\newcommand{\bichi}{\boldsymbol{\chi}}\newcommand{\bipsi}{\boldsymbol{\psi}}\newcommand{\biomega}{\boldsymbol{\omega}}{L_1}$ is 1 for happy-adapted trials and 0 otherwise, $\newcommand{\bialpha}{\boldsymbol{\alpha}}\newcommand{\bibeta}{\boldsymbol{\beta}}\newcommand{\bigamma}{\boldsymbol{\gamma}}\newcommand{\bidelta}{\boldsymbol{\delta}}\newcommand{\bivarepsilon}{\boldsymbol{\varepsilon}}\newcommand{\bizeta}{\boldsymbol{\zeta}}\newcommand{\bieta}{\boldsymbol{\eta}}\newcommand{\bitheta}{\boldsymbol{\theta}}\newcommand{\biiota}{\boldsymbol{\iota}}\newcommand{\bikappa}{\boldsymbol{\kappa}}\newcommand{\bilambda}{\boldsymbol{\lambda}}\newcommand{\bimu}{\boldsymbol{\mu}}\newcommand{\binu}{\boldsymbol{\nu}}\newcommand{\bixi}{\boldsymbol{\xi}}\newcommand{\biomicron}{\boldsymbol{\micron}}\newcommand{\bipi}{\boldsymbol{\pi}}\newcommand{\birho}{\boldsymbol{\rho}}\newcommand{\bisigma}{\boldsymbol{\sigma}}\newcommand{\bitau}{\boldsymbol{\tau}}\newcommand{\biupsilon}{\boldsymbol{\upsilon}}\newcommand{\biphi}{\boldsymbol{\phi}}\newcommand{\bichi}{\boldsymbol{\chi}}\newcommand{\bipsi}{\boldsymbol{\psi}}\newcommand{\biomega}{\boldsymbol{\omega}}{L_2}$ is 1 for sad-adapted trials and 0 otherwise, and $\newcommand{\bialpha}{\boldsymbol{\alpha}}\newcommand{\bibeta}{\boldsymbol{\beta}}\newcommand{\bigamma}{\boldsymbol{\gamma}}\newcommand{\bidelta}{\boldsymbol{\delta}}\newcommand{\bivarepsilon}{\boldsymbol{\varepsilon}}\newcommand{\bizeta}{\boldsymbol{\zeta}}\newcommand{\bieta}{\boldsymbol{\eta}}\newcommand{\bitheta}{\boldsymbol{\theta}}\newcommand{\biiota}{\boldsymbol{\iota}}\newcommand{\bikappa}{\boldsymbol{\kappa}}\newcommand{\bilambda}{\boldsymbol{\lambda}}\newcommand{\bimu}{\boldsymbol{\mu}}\newcommand{\binu}{\boldsymbol{\nu}}\newcommand{\bixi}{\boldsymbol{\xi}}\newcommand{\biomicron}{\boldsymbol{\micron}}\newcommand{\bipi}{\boldsymbol{\pi}}\newcommand{\birho}{\boldsymbol{\rho}}\newcommand{\bisigma}{\boldsymbol{\sigma}}\newcommand{\bitau}{\boldsymbol{\tau}}\newcommand{\biupsilon}{\boldsymbol{\upsilon}}\newcommand{\biphi}{\boldsymbol{\phi}}\newcommand{\bichi}{\boldsymbol{\chi}}\newcommand{\bipsi}{\boldsymbol{\psi}}\newcommand{\biomega}{\boldsymbol{\omega}}{L_3}$ is 1 for either happy- or sad-adapted trials and 0 otherwise. $\newcommand{\bialpha}{\boldsymbol{\alpha}}\newcommand{\bibeta}{\boldsymbol{\beta}}\newcommand{\bigamma}{\boldsymbol{\gamma}}\newcommand{\bidelta}{\boldsymbol{\delta}}\newcommand{\bivarepsilon}{\boldsymbol{\varepsilon}}\newcommand{\bizeta}{\boldsymbol{\zeta}}\newcommand{\bieta}{\boldsymbol{\eta}}\newcommand{\bitheta}{\boldsymbol{\theta}}\newcommand{\biiota}{\boldsymbol{\iota}}\newcommand{\bikappa}{\boldsymbol{\kappa}}\newcommand{\bilambda}{\boldsymbol{\lambda}}\newcommand{\bimu}{\boldsymbol{\mu}}\newcommand{\binu}{\boldsymbol{\nu}}\newcommand{\bixi}{\boldsymbol{\xi}}\newcommand{\biomicron}{\boldsymbol{\micron}}\newcommand{\bipi}{\boldsymbol{\pi}}\newcommand{\birho}{\boldsymbol{\rho}}\newcommand{\bisigma}{\boldsymbol{\sigma}}\newcommand{\bitau}{\boldsymbol{\tau}}\newcommand{\biupsilon}{\boldsymbol{\upsilon}}\newcommand{\biphi}{\boldsymbol{\phi}}\newcommand{\bichi}{\boldsymbol{\chi}}\newcommand{\bipsi}{\boldsymbol{\psi}}\newcommand{\biomega}{\boldsymbol{\omega}}{\beta _1}$ accounts for a bias in the unadapted condition, and $\newcommand{\bialpha}{\boldsymbol{\alpha}}\newcommand{\bibeta}{\boldsymbol{\beta}}\newcommand{\bigamma}{\boldsymbol{\gamma}}\newcommand{\bidelta}{\boldsymbol{\delta}}\newcommand{\bivarepsilon}{\boldsymbol{\varepsilon}}\newcommand{\bizeta}{\boldsymbol{\zeta}}\newcommand{\bieta}{\boldsymbol{\eta}}\newcommand{\bitheta}{\boldsymbol{\theta}}\newcommand{\biiota}{\boldsymbol{\iota}}\newcommand{\bikappa}{\boldsymbol{\kappa}}\newcommand{\bilambda}{\boldsymbol{\lambda}}\newcommand{\bimu}{\boldsymbol{\mu}}\newcommand{\binu}{\boldsymbol{\nu}}\newcommand{\bixi}{\boldsymbol{\xi}}\newcommand{\biomicron}{\boldsymbol{\micron}}\newcommand{\bipi}{\boldsymbol{\pi}}\newcommand{\birho}{\boldsymbol{\rho}}\newcommand{\bisigma}{\boldsymbol{\sigma}}\newcommand{\bitau}{\boldsymbol{\tau}}\newcommand{\biupsilon}{\boldsymbol{\upsilon}}\newcommand{\biphi}{\boldsymbol{\phi}}\newcommand{\bichi}{\boldsymbol{\chi}}\newcommand{\bipsi}{\boldsymbol{\psi}}\newcommand{\biomega}{\boldsymbol{\omega}}{\beta _2}$ and $\newcommand{\bialpha}{\boldsymbol{\alpha}}\newcommand{\bibeta}{\boldsymbol{\beta}}\newcommand{\bigamma}{\boldsymbol{\gamma}}\newcommand{\bidelta}{\boldsymbol{\delta}}\newcommand{\bivarepsilon}{\boldsymbol{\varepsilon}}\newcommand{\bizeta}{\boldsymbol{\zeta}}\newcommand{\bieta}{\boldsymbol{\eta}}\newcommand{\bitheta}{\boldsymbol{\theta}}\newcommand{\biiota}{\boldsymbol{\iota}}\newcommand{\bikappa}{\boldsymbol{\kappa}}\newcommand{\bilambda}{\boldsymbol{\lambda}}\newcommand{\bimu}{\boldsymbol{\mu}}\newcommand{\binu}{\boldsymbol{\nu}}\newcommand{\bixi}{\boldsymbol{\xi}}\newcommand{\biomicron}{\boldsymbol{\micron}}\newcommand{\bipi}{\boldsymbol{\pi}}\newcommand{\birho}{\boldsymbol{\rho}}\newcommand{\bisigma}{\boldsymbol{\sigma}}\newcommand{\bitau}{\boldsymbol{\tau}}\newcommand{\biupsilon}{\boldsymbol{\upsilon}}\newcommand{\biphi}{\boldsymbol{\phi}}\newcommand{\bichi}{\boldsymbol{\chi}}\newcommand{\bipsi}{\boldsymbol{\psi}}\newcommand{\biomega}{\boldsymbol{\omega}}{\beta _3}$ indicate the change of PSEs in the adapted conditions compared to the unadapted condition. $\newcommand{\bialpha}{\boldsymbol{\alpha}}\newcommand{\bibeta}{\boldsymbol{\beta}}\newcommand{\bigamma}{\boldsymbol{\gamma}}\newcommand{\bidelta}{\boldsymbol{\delta}}\newcommand{\bivarepsilon}{\boldsymbol{\varepsilon}}\newcommand{\bizeta}{\boldsymbol{\zeta}}\newcommand{\bieta}{\boldsymbol{\eta}}\newcommand{\bitheta}{\boldsymbol{\theta}}\newcommand{\biiota}{\boldsymbol{\iota}}\newcommand{\bikappa}{\boldsymbol{\kappa}}\newcommand{\bilambda}{\boldsymbol{\lambda}}\newcommand{\bimu}{\boldsymbol{\mu}}\newcommand{\binu}{\boldsymbol{\nu}}\newcommand{\bixi}{\boldsymbol{\xi}}\newcommand{\biomicron}{\boldsymbol{\micron}}\newcommand{\bipi}{\boldsymbol{\pi}}\newcommand{\birho}{\boldsymbol{\rho}}\newcommand{\bisigma}{\boldsymbol{\sigma}}\newcommand{\bitau}{\boldsymbol{\tau}}\newcommand{\biupsilon}{\boldsymbol{\upsilon}}\newcommand{\biphi}{\boldsymbol{\phi}}\newcommand{\bichi}{\boldsymbol{\chi}}\newcommand{\bipsi}{\boldsymbol{\psi}}\newcommand{\biomega}{\boldsymbol{\omega}}{\beta _4}$ indicates the change in the slope of the adapted psychometric functions compared to the unadapted condition.

#### Drift-diffusion models

We modeled the full set of data—the distribution of response times and choices—assuming that decisions in our task were made by accumulating noisy sensory evidence toward a threshold (decision bound). A simplified version of this bounded accumulation process is formalized by drift-diffusion models, which have been shown to successfully explain choice and response time for a broad range of cognitive and perceptual decision-making tasks (Krajbich, Armel, & Rangel, 2010; Purcell et al., 2010; Shadlen & Kiani, 2013; Smith & Ratcliff, 2004). The visual stimulus gives rise to a sensory representation that fluctuates stochastically over time. The evidence conferred by these momentary representations are modeled as draws from a unit-variance Gaussian distribution, whose mean is a monotonic function of the stimulus strength. The momentary evidence is accumulated until one of the two decision bounds is reached (upper bound for sad responses and lower bound for happy responses). The time to bound is decision time. The experimentally measured response time on each trial is the sum of decision time and some nondecision time, which is comprised by sensory and motor delays.

Due to the stochastic nature of evidence, integration of evidence is a diffusion process with drift, where the average drift rate is the mean of momentary evidence. Larger drift toward a decision bound increases the probability of making the corresponding choice and reduces its decision time. If adaptation changes sensory representations to shift the drift rates in favor of one of the choices, it will cause a shift in the psychometric function without changing its slope. Also, changes in drift rates will cause an equal shift in the chronometric function (response times as a function of stimulus strength).

The height of decision bounds also influences the likelihood of choices and their decision times. Specifically, smaller bound heights are associated with shorter decision times and higher susceptibility to the noise of the diffusion process. This increased susceptibility to noise translates into a lower slope in the psychometric function. An asymmetry in the height of the two decision bounds causes a shift in the psychometric function by increasing the likelihood of the choice associated with the smaller bound. There will also be a shift in chronometric functions. However, unlike changes of drift rate, this shift is accompanied by a split in the chronometric function, where for each stimulus strength, responses associated with the smaller bound are faster.

We used two methods to fit the drift-diffusion model to the data. We report the results for the data combined across participants, but similar, albeit noisier, results are obtained from single subject fits.

**Figure 3:**
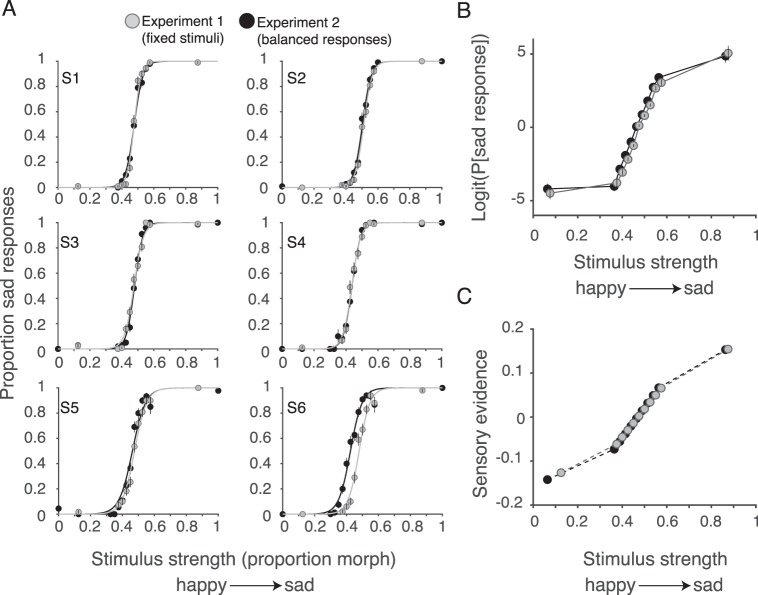
Perceptual validity of the stimulus set. The unadapted condition was used to assess the validity of the stimulus set. (A) Psychometric functions from Experiments 1 (fixed stimulus set) and 2 (balanced responses) show smooth and generally monotonic patterns for each subject. (B) The psychometric functions fit to the pooled data across observers were plotted using the logit function on the y-axis rather than percent correct. If stimuli that are equally spaced on the x-axis are equally spaced perceptually, and the participant makes a decision via a diffusion process with fixed bounds, then a linear relationship is predicted. This approximately holds for the middle range of stimuli in both experiments. Linearity fails for the extreme stimuli. (C) The level of sensory evidence for each stimulus was computed from a diffusion process in which the bounds could be asymmetric. The results again show a linear relationship for the middle stimuli in both experiments.

Our first fitting method aimed at understanding how sensory evidence changed with the stimulus strength. There is no a priori reason to assume that a linear morph line in the stimulus space should lead to a linear change of sensory evidence. Therefore, we set out to elucidate this relationship using both model-free and model-based methods. For the model-free method, we calculated the probability (Figure 3A) and the log odds of the probability (Figure 3B) of sad choices for each stimulus strength. Changes of the *logit*[*P*(*sad*)] as a function of stimulus strength approximate changes in the average evidence conferred by the stimuli (Kiani, Cueva, Reppas, & Newsome, 2014). For the model-based approach, we created a high-parameter drift-diffusion model with 1 degree of freedom (*df*) for the drift rate of each stimulus strength (Figure 3C). Overall, the model had 14 parameters: 11 parameters for the sensitivities (drift rates) of the 11 stimuli; two parameters for the decision boundaries (happy and sad); and one parameter for nondecision time. Fitting such a model to data is computationally intensive because the number of model evaluations for convergence of the fitting procedure grows rapidly with the number of model parameters. Also, avoiding local minima in a high-parameter model requires repeating the fitting process from numerous starting points, which multiplicatively increases the number of model evaluations. To reduce computational costs, we relied on a fast fitting process developed by Palmer, Huk, and Shadlen (2005) that uses analytical solutions of the drift-diffusion model for the probability of choices and the mean reaction time for each stimulus strength (Link, 1992). Briefly, for a drift-diffusion process that starts at zero:

\newcommand{\bialpha}{\boldsymbol{\alpha}}\newcommand{\bibeta}{\boldsymbol{\beta}}\newcommand{\bigamma}{\boldsymbol{\gamma}}\newcommand{\bidelta}{\boldsymbol{\delta}}\newcommand{\bivarepsilon}{\boldsymbol{\varepsilon}}\newcommand{\bizeta}{\boldsymbol{\zeta}}\newcommand{\bieta}{\boldsymbol{\eta}}\newcommand{\bitheta}{\boldsymbol{\theta}}\newcommand{\biiota}{\boldsymbol{\iota}}\newcommand{\bikappa}{\boldsymbol{\kappa}}\newcommand{\bilambda}{\boldsymbol{\lambda}}\newcommand{\bimu}{\boldsymbol{\mu}}\newcommand{\binu}{\boldsymbol{\nu}}\newcommand{\bixi}{\boldsymbol{\xi}}\newcommand{\biomicron}{\boldsymbol{\micron}}\newcommand{\bipi}{\boldsymbol{\pi}}\newcommand{\birho}{\boldsymbol{\rho}}\newcommand{\bisigma}{\boldsymbol{\sigma}}\newcommand{\bitau}{\boldsymbol{\tau}}\newcommand{\biupsilon}{\boldsymbol{\upsilon}}\newcommand{\biphi}{\boldsymbol{\phi}}\newcommand{\bichi}{\boldsymbol{\chi}}\newcommand{\bipsi}{\boldsymbol{\psi}}\newcommand{\biomega}{\boldsymbol{\omega}}\begin{equation}\tag{3}P\left( {sad|S} \right) = {{exp\left( {2{\mu _S}\bar B} \right) - exp\left( { - 2{\mu _S}\Delta B} \right)} \over {exp\left( {2{\mu _S}\bar B} \right) - exp\left( { - 2{\mu _S}\bar B} \right)}}\end{equation}

\newcommand{\bialpha}{\boldsymbol{\alpha}}\newcommand{\bibeta}{\boldsymbol{\beta}}\newcommand{\bigamma}{\boldsymbol{\gamma}}\newcommand{\bidelta}{\boldsymbol{\delta}}\newcommand{\bivarepsilon}{\boldsymbol{\varepsilon}}\newcommand{\bizeta}{\boldsymbol{\zeta}}\newcommand{\bieta}{\boldsymbol{\eta}}\newcommand{\bitheta}{\boldsymbol{\theta}}\newcommand{\biiota}{\boldsymbol{\iota}}\newcommand{\bikappa}{\boldsymbol{\kappa}}\newcommand{\bilambda}{\boldsymbol{\lambda}}\newcommand{\bimu}{\boldsymbol{\mu}}\newcommand{\binu}{\boldsymbol{\nu}}\newcommand{\bixi}{\boldsymbol{\xi}}\newcommand{\biomicron}{\boldsymbol{\micron}}\newcommand{\bipi}{\boldsymbol{\pi}}\newcommand{\birho}{\boldsymbol{\rho}}\newcommand{\bisigma}{\boldsymbol{\sigma}}\newcommand{\bitau}{\boldsymbol{\tau}}\newcommand{\biupsilon}{\boldsymbol{\upsilon}}\newcommand{\biphi}{\boldsymbol{\phi}}\newcommand{\bichi}{\boldsymbol{\chi}}\newcommand{\bipsi}{\boldsymbol{\psi}}\newcommand{\biomega}{\boldsymbol{\omega}}\begin{equation}\tag{4}\bar T\left( S \right) = {{\bar B\left( {2P\left( {sad|S} \right) - 1} \right) - \Delta B} \over {\mu _S}} + \overline {T_0} \end{equation}

where $\newcommand{\bialpha}{\boldsymbol{\alpha}}\newcommand{\bibeta}{\boldsymbol{\beta}}\newcommand{\bigamma}{\boldsymbol{\gamma}}\newcommand{\bidelta}{\boldsymbol{\delta}}\newcommand{\bivarepsilon}{\boldsymbol{\varepsilon}}\newcommand{\bizeta}{\boldsymbol{\zeta}}\newcommand{\bieta}{\boldsymbol{\eta}}\newcommand{\bitheta}{\boldsymbol{\theta}}\newcommand{\biiota}{\boldsymbol{\iota}}\newcommand{\bikappa}{\boldsymbol{\kappa}}\newcommand{\bilambda}{\boldsymbol{\lambda}}\newcommand{\bimu}{\boldsymbol{\mu}}\newcommand{\binu}{\boldsymbol{\nu}}\newcommand{\bixi}{\boldsymbol{\xi}}\newcommand{\biomicron}{\boldsymbol{\micron}}\newcommand{\bipi}{\boldsymbol{\pi}}\newcommand{\birho}{\boldsymbol{\rho}}\newcommand{\bisigma}{\boldsymbol{\sigma}}\newcommand{\bitau}{\boldsymbol{\tau}}\newcommand{\biupsilon}{\boldsymbol{\upsilon}}\newcommand{\biphi}{\boldsymbol{\phi}}\newcommand{\bichi}{\boldsymbol{\chi}}\newcommand{\bipsi}{\boldsymbol{\psi}}\newcommand{\biomega}{\boldsymbol{\omega}}P\left( {sad|S} \right)$ and $\newcommand{\bialpha}{\boldsymbol{\alpha}}\newcommand{\bibeta}{\boldsymbol{\beta}}\newcommand{\bigamma}{\boldsymbol{\gamma}}\newcommand{\bidelta}{\boldsymbol{\delta}}\newcommand{\bivarepsilon}{\boldsymbol{\varepsilon}}\newcommand{\bizeta}{\boldsymbol{\zeta}}\newcommand{\bieta}{\boldsymbol{\eta}}\newcommand{\bitheta}{\boldsymbol{\theta}}\newcommand{\biiota}{\boldsymbol{\iota}}\newcommand{\bikappa}{\boldsymbol{\kappa}}\newcommand{\bilambda}{\boldsymbol{\lambda}}\newcommand{\bimu}{\boldsymbol{\mu}}\newcommand{\binu}{\boldsymbol{\nu}}\newcommand{\bixi}{\boldsymbol{\xi}}\newcommand{\biomicron}{\boldsymbol{\micron}}\newcommand{\bipi}{\boldsymbol{\pi}}\newcommand{\birho}{\boldsymbol{\rho}}\newcommand{\bisigma}{\boldsymbol{\sigma}}\newcommand{\bitau}{\boldsymbol{\tau}}\newcommand{\biupsilon}{\boldsymbol{\upsilon}}\newcommand{\biphi}{\boldsymbol{\phi}}\newcommand{\bichi}{\boldsymbol{\chi}}\newcommand{\bipsi}{\boldsymbol{\psi}}\newcommand{\biomega}{\boldsymbol{\omega}}\bar T\left( S \right)$ are the probability of making sad responses and the mean reaction time for stimulus $\newcommand{\bialpha}{\boldsymbol{\alpha}}\newcommand{\bibeta}{\boldsymbol{\beta}}\newcommand{\bigamma}{\boldsymbol{\gamma}}\newcommand{\bidelta}{\boldsymbol{\delta}}\newcommand{\bivarepsilon}{\boldsymbol{\varepsilon}}\newcommand{\bizeta}{\boldsymbol{\zeta}}\newcommand{\bieta}{\boldsymbol{\eta}}\newcommand{\bitheta}{\boldsymbol{\theta}}\newcommand{\biiota}{\boldsymbol{\iota}}\newcommand{\bikappa}{\boldsymbol{\kappa}}\newcommand{\bilambda}{\boldsymbol{\lambda}}\newcommand{\bimu}{\boldsymbol{\mu}}\newcommand{\binu}{\boldsymbol{\nu}}\newcommand{\bixi}{\boldsymbol{\xi}}\newcommand{\biomicron}{\boldsymbol{\micron}}\newcommand{\bipi}{\boldsymbol{\pi}}\newcommand{\birho}{\boldsymbol{\rho}}\newcommand{\bisigma}{\boldsymbol{\sigma}}\newcommand{\bitau}{\boldsymbol{\tau}}\newcommand{\biupsilon}{\boldsymbol{\upsilon}}\newcommand{\biphi}{\boldsymbol{\phi}}\newcommand{\bichi}{\boldsymbol{\chi}}\newcommand{\bipsi}{\boldsymbol{\psi}}\newcommand{\biomega}{\boldsymbol{\omega}}S$. $\newcommand{\bialpha}{\boldsymbol{\alpha}}\newcommand{\bibeta}{\boldsymbol{\beta}}\newcommand{\bigamma}{\boldsymbol{\gamma}}\newcommand{\bidelta}{\boldsymbol{\delta}}\newcommand{\bivarepsilon}{\boldsymbol{\varepsilon}}\newcommand{\bizeta}{\boldsymbol{\zeta}}\newcommand{\bieta}{\boldsymbol{\eta}}\newcommand{\bitheta}{\boldsymbol{\theta}}\newcommand{\biiota}{\boldsymbol{\iota}}\newcommand{\bikappa}{\boldsymbol{\kappa}}\newcommand{\bilambda}{\boldsymbol{\lambda}}\newcommand{\bimu}{\boldsymbol{\mu}}\newcommand{\binu}{\boldsymbol{\nu}}\newcommand{\bixi}{\boldsymbol{\xi}}\newcommand{\biomicron}{\boldsymbol{\micron}}\newcommand{\bipi}{\boldsymbol{\pi}}\newcommand{\birho}{\boldsymbol{\rho}}\newcommand{\bisigma}{\boldsymbol{\sigma}}\newcommand{\bitau}{\boldsymbol{\tau}}\newcommand{\biupsilon}{\boldsymbol{\upsilon}}\newcommand{\biphi}{\boldsymbol{\phi}}\newcommand{\bichi}{\boldsymbol{\chi}}\newcommand{\bipsi}{\boldsymbol{\psi}}\newcommand{\biomega}{\boldsymbol{\omega}}\bar B$ is the average of the absolute bound heights for the two choices, $\newcommand{\bialpha}{\boldsymbol{\alpha}}\newcommand{\bibeta}{\boldsymbol{\beta}}\newcommand{\bigamma}{\boldsymbol{\gamma}}\newcommand{\bidelta}{\boldsymbol{\delta}}\newcommand{\bivarepsilon}{\boldsymbol{\varepsilon}}\newcommand{\bizeta}{\boldsymbol{\zeta}}\newcommand{\bieta}{\boldsymbol{\eta}}\newcommand{\bitheta}{\boldsymbol{\theta}}\newcommand{\biiota}{\boldsymbol{\iota}}\newcommand{\bikappa}{\boldsymbol{\kappa}}\newcommand{\bilambda}{\boldsymbol{\lambda}}\newcommand{\bimu}{\boldsymbol{\mu}}\newcommand{\binu}{\boldsymbol{\nu}}\newcommand{\bixi}{\boldsymbol{\xi}}\newcommand{\biomicron}{\boldsymbol{\micron}}\newcommand{\bipi}{\boldsymbol{\pi}}\newcommand{\birho}{\boldsymbol{\rho}}\newcommand{\bisigma}{\boldsymbol{\sigma}}\newcommand{\bitau}{\boldsymbol{\tau}}\newcommand{\biupsilon}{\boldsymbol{\upsilon}}\newcommand{\biphi}{\boldsymbol{\phi}}\newcommand{\bichi}{\boldsymbol{\chi}}\newcommand{\bipsi}{\boldsymbol{\psi}}\newcommand{\biomega}{\boldsymbol{\omega}}\Delta B$ is the offset of the absolute bound heights, $\newcommand{\bialpha}{\boldsymbol{\alpha}}\newcommand{\bibeta}{\boldsymbol{\beta}}\newcommand{\bigamma}{\boldsymbol{\gamma}}\newcommand{\bidelta}{\boldsymbol{\delta}}\newcommand{\bivarepsilon}{\boldsymbol{\varepsilon}}\newcommand{\bizeta}{\boldsymbol{\zeta}}\newcommand{\bieta}{\boldsymbol{\eta}}\newcommand{\bitheta}{\boldsymbol{\theta}}\newcommand{\biiota}{\boldsymbol{\iota}}\newcommand{\bikappa}{\boldsymbol{\kappa}}\newcommand{\bilambda}{\boldsymbol{\lambda}}\newcommand{\bimu}{\boldsymbol{\mu}}\newcommand{\binu}{\boldsymbol{\nu}}\newcommand{\bixi}{\boldsymbol{\xi}}\newcommand{\biomicron}{\boldsymbol{\micron}}\newcommand{\bipi}{\boldsymbol{\pi}}\newcommand{\birho}{\boldsymbol{\rho}}\newcommand{\bisigma}{\boldsymbol{\sigma}}\newcommand{\bitau}{\boldsymbol{\tau}}\newcommand{\biupsilon}{\boldsymbol{\upsilon}}\newcommand{\biphi}{\boldsymbol{\phi}}\newcommand{\bichi}{\boldsymbol{\chi}}\newcommand{\bipsi}{\boldsymbol{\psi}}\newcommand{\biomega}{\boldsymbol{\omega}}{\mu _S}$ is the drift rate associated with stimulus $\newcommand{\bialpha}{\boldsymbol{\alpha}}\newcommand{\bibeta}{\boldsymbol{\beta}}\newcommand{\bigamma}{\boldsymbol{\gamma}}\newcommand{\bidelta}{\boldsymbol{\delta}}\newcommand{\bivarepsilon}{\boldsymbol{\varepsilon}}\newcommand{\bizeta}{\boldsymbol{\zeta}}\newcommand{\bieta}{\boldsymbol{\eta}}\newcommand{\bitheta}{\boldsymbol{\theta}}\newcommand{\biiota}{\boldsymbol{\iota}}\newcommand{\bikappa}{\boldsymbol{\kappa}}\newcommand{\bilambda}{\boldsymbol{\lambda}}\newcommand{\bimu}{\boldsymbol{\mu}}\newcommand{\binu}{\boldsymbol{\nu}}\newcommand{\bixi}{\boldsymbol{\xi}}\newcommand{\biomicron}{\boldsymbol{\micron}}\newcommand{\bipi}{\boldsymbol{\pi}}\newcommand{\birho}{\boldsymbol{\rho}}\newcommand{\bisigma}{\boldsymbol{\sigma}}\newcommand{\bitau}{\boldsymbol{\tau}}\newcommand{\biupsilon}{\boldsymbol{\upsilon}}\newcommand{\biphi}{\boldsymbol{\phi}}\newcommand{\bichi}{\boldsymbol{\chi}}\newcommand{\bipsi}{\boldsymbol{\psi}}\newcommand{\biomega}{\boldsymbol{\omega}}S$, and $\newcommand{\bialpha}{\boldsymbol{\alpha}}\newcommand{\bibeta}{\boldsymbol{\beta}}\newcommand{\bigamma}{\boldsymbol{\gamma}}\newcommand{\bidelta}{\boldsymbol{\delta}}\newcommand{\bivarepsilon}{\boldsymbol{\varepsilon}}\newcommand{\bizeta}{\boldsymbol{\zeta}}\newcommand{\bieta}{\boldsymbol{\eta}}\newcommand{\bitheta}{\boldsymbol{\theta}}\newcommand{\biiota}{\boldsymbol{\iota}}\newcommand{\bikappa}{\boldsymbol{\kappa}}\newcommand{\bilambda}{\boldsymbol{\lambda}}\newcommand{\bimu}{\boldsymbol{\mu}}\newcommand{\binu}{\boldsymbol{\nu}}\newcommand{\bixi}{\boldsymbol{\xi}}\newcommand{\biomicron}{\boldsymbol{\micron}}\newcommand{\bipi}{\boldsymbol{\pi}}\newcommand{\birho}{\boldsymbol{\rho}}\newcommand{\bisigma}{\boldsymbol{\sigma}}\newcommand{\bitau}{\boldsymbol{\tau}}\newcommand{\biupsilon}{\boldsymbol{\upsilon}}\newcommand{\biphi}{\boldsymbol{\phi}}\newcommand{\bichi}{\boldsymbol{\chi}}\newcommand{\bipsi}{\boldsymbol{\psi}}\newcommand{\biomega}{\boldsymbol{\omega}}\overline {T_0}$ is the average nondecision time. Using these analytical solutions, we optimized the model parameters to maximize the sum of the log likelihood of observed choices and the log likelihood of observed response times (see Palmer et al., 2005 for details). This fitting procedure is fast and suitable for high-parameter models, but it does not optimize the joint likelihood of choices and response times on single trials (see below for a better alternative). Both the model-based and model-free approaches indicated a linear change of drift rates with stimulus strength for the intermediate stimuli but not for the extreme stimuli. We used this knowledge to develop a more accurate but computationally more intensive fitting procedure.

Our second fitting method aimed at providing a precise estimation of changes of the drift rates, bound heights, and nondecision time across different adaptation conditions. It calculated the probability density of different response times for each choice and found model parameters that maximized the joint likelihood of experimentally observed choices and response times across trials. The probability of crossing the upper and lower decision bounds at each decision time was calculated by solving the Fokker–Planck equation (Karlin & Taylor, 1981; Kiani & Shadlen, 2009; Purcell & Kiani, 2016a):

\newcommand{\bialpha}{\boldsymbol{\alpha}}\newcommand{\bibeta}{\boldsymbol{\beta}}\newcommand{\bigamma}{\boldsymbol{\gamma}}\newcommand{\bidelta}{\boldsymbol{\delta}}\newcommand{\bivarepsilon}{\boldsymbol{\varepsilon}}\newcommand{\bizeta}{\boldsymbol{\zeta}}\newcommand{\bieta}{\boldsymbol{\eta}}\newcommand{\bitheta}{\boldsymbol{\theta}}\newcommand{\biiota}{\boldsymbol{\iota}}\newcommand{\bikappa}{\boldsymbol{\kappa}}\newcommand{\bilambda}{\boldsymbol{\lambda}}\newcommand{\bimu}{\boldsymbol{\mu}}\newcommand{\binu}{\boldsymbol{\nu}}\newcommand{\bixi}{\boldsymbol{\xi}}\newcommand{\biomicron}{\boldsymbol{\micron}}\newcommand{\bipi}{\boldsymbol{\pi}}\newcommand{\birho}{\boldsymbol{\rho}}\newcommand{\bisigma}{\boldsymbol{\sigma}}\newcommand{\bitau}{\boldsymbol{\tau}}\newcommand{\biupsilon}{\boldsymbol{\upsilon}}\newcommand{\biphi}{\boldsymbol{\phi}}\newcommand{\bichi}{\boldsymbol{\chi}}\newcommand{\bipsi}{\boldsymbol{\psi}}\newcommand{\biomega}{\boldsymbol{\omega}}\begin{equation}\tag{5}{{\partial p\left( {v,t} \right)} \over {\partial t}} = - {\mu _S}{{\partial p\left( {v,t} \right)} \over {\partial v}} + 0.5{{{\partial ^2}p\left( {v,t} \right)} \over {\partial {v^2}}}\end{equation}

where $\newcommand{\bialpha}{\boldsymbol{\alpha}}\newcommand{\bibeta}{\boldsymbol{\beta}}\newcommand{\bigamma}{\boldsymbol{\gamma}}\newcommand{\bidelta}{\boldsymbol{\delta}}\newcommand{\bivarepsilon}{\boldsymbol{\varepsilon}}\newcommand{\bizeta}{\boldsymbol{\zeta}}\newcommand{\bieta}{\boldsymbol{\eta}}\newcommand{\bitheta}{\boldsymbol{\theta}}\newcommand{\biiota}{\boldsymbol{\iota}}\newcommand{\bikappa}{\boldsymbol{\kappa}}\newcommand{\bilambda}{\boldsymbol{\lambda}}\newcommand{\bimu}{\boldsymbol{\mu}}\newcommand{\binu}{\boldsymbol{\nu}}\newcommand{\bixi}{\boldsymbol{\xi}}\newcommand{\biomicron}{\boldsymbol{\micron}}\newcommand{\bipi}{\boldsymbol{\pi}}\newcommand{\birho}{\boldsymbol{\rho}}\newcommand{\bisigma}{\boldsymbol{\sigma}}\newcommand{\bitau}{\boldsymbol{\tau}}\newcommand{\biupsilon}{\boldsymbol{\upsilon}}\newcommand{\biphi}{\boldsymbol{\phi}}\newcommand{\bichi}{\boldsymbol{\chi}}\newcommand{\bipsi}{\boldsymbol{\psi}}\newcommand{\biomega}{\boldsymbol{\omega}}v$ is the accumulated evidence and $\newcommand{\bialpha}{\boldsymbol{\alpha}}\newcommand{\bibeta}{\boldsymbol{\beta}}\newcommand{\bigamma}{\boldsymbol{\gamma}}\newcommand{\bidelta}{\boldsymbol{\delta}}\newcommand{\bivarepsilon}{\boldsymbol{\varepsilon}}\newcommand{\bizeta}{\boldsymbol{\zeta}}\newcommand{\bieta}{\boldsymbol{\eta}}\newcommand{\bitheta}{\boldsymbol{\theta}}\newcommand{\biiota}{\boldsymbol{\iota}}\newcommand{\bikappa}{\boldsymbol{\kappa}}\newcommand{\bilambda}{\boldsymbol{\lambda}}\newcommand{\bimu}{\boldsymbol{\mu}}\newcommand{\binu}{\boldsymbol{\nu}}\newcommand{\bixi}{\boldsymbol{\xi}}\newcommand{\biomicron}{\boldsymbol{\micron}}\newcommand{\bipi}{\boldsymbol{\pi}}\newcommand{\birho}{\boldsymbol{\rho}}\newcommand{\bisigma}{\boldsymbol{\sigma}}\newcommand{\bitau}{\boldsymbol{\tau}}\newcommand{\biupsilon}{\boldsymbol{\upsilon}}\newcommand{\biphi}{\boldsymbol{\phi}}\newcommand{\bichi}{\boldsymbol{\chi}}\newcommand{\bipsi}{\boldsymbol{\psi}}\newcommand{\biomega}{\boldsymbol{\omega}}p\left( {v,t} \right)$ is the probability density of the accumulated evidence between the two decision bounds at time $\newcommand{\bialpha}{\boldsymbol{\alpha}}\newcommand{\bibeta}{\boldsymbol{\beta}}\newcommand{\bigamma}{\boldsymbol{\gamma}}\newcommand{\bidelta}{\boldsymbol{\delta}}\newcommand{\bivarepsilon}{\boldsymbol{\varepsilon}}\newcommand{\bizeta}{\boldsymbol{\zeta}}\newcommand{\bieta}{\boldsymbol{\eta}}\newcommand{\bitheta}{\boldsymbol{\theta}}\newcommand{\biiota}{\boldsymbol{\iota}}\newcommand{\bikappa}{\boldsymbol{\kappa}}\newcommand{\bilambda}{\boldsymbol{\lambda}}\newcommand{\bimu}{\boldsymbol{\mu}}\newcommand{\binu}{\boldsymbol{\nu}}\newcommand{\bixi}{\boldsymbol{\xi}}\newcommand{\biomicron}{\boldsymbol{\micron}}\newcommand{\bipi}{\boldsymbol{\pi}}\newcommand{\birho}{\boldsymbol{\rho}}\newcommand{\bisigma}{\boldsymbol{\sigma}}\newcommand{\bitau}{\boldsymbol{\tau}}\newcommand{\biupsilon}{\boldsymbol{\upsilon}}\newcommand{\biphi}{\boldsymbol{\phi}}\newcommand{\bichi}{\boldsymbol{\chi}}\newcommand{\bipsi}{\boldsymbol{\psi}}\newcommand{\biomega}{\boldsymbol{\omega}}t$. The boundary conditions of Equation 5 are:

\newcommand{\bialpha}{\boldsymbol{\alpha}}\newcommand{\bibeta}{\boldsymbol{\beta}}\newcommand{\bigamma}{\boldsymbol{\gamma}}\newcommand{\bidelta}{\boldsymbol{\delta}}\newcommand{\bivarepsilon}{\boldsymbol{\varepsilon}}\newcommand{\bizeta}{\boldsymbol{\zeta}}\newcommand{\bieta}{\boldsymbol{\eta}}\newcommand{\bitheta}{\boldsymbol{\theta}}\newcommand{\biiota}{\boldsymbol{\iota}}\newcommand{\bikappa}{\boldsymbol{\kappa}}\newcommand{\bilambda}{\boldsymbol{\lambda}}\newcommand{\bimu}{\boldsymbol{\mu}}\newcommand{\binu}{\boldsymbol{\nu}}\newcommand{\bixi}{\boldsymbol{\xi}}\newcommand{\biomicron}{\boldsymbol{\micron}}\newcommand{\bipi}{\boldsymbol{\pi}}\newcommand{\birho}{\boldsymbol{\rho}}\newcommand{\bisigma}{\boldsymbol{\sigma}}\newcommand{\bitau}{\boldsymbol{\tau}}\newcommand{\biupsilon}{\boldsymbol{\upsilon}}\newcommand{\biphi}{\boldsymbol{\phi}}\newcommand{\bichi}{\boldsymbol{\chi}}\newcommand{\bipsi}{\boldsymbol{\psi}}\newcommand{\biomega}{\boldsymbol{\omega}}\begin{equation}\tag{6}p\left( {v,0} \right) = \delta \left( v \right)\end{equation}

\newcommand{\bialpha}{\boldsymbol{\alpha}}\newcommand{\bibeta}{\boldsymbol{\beta}}\newcommand{\bigamma}{\boldsymbol{\gamma}}\newcommand{\bidelta}{\boldsymbol{\delta}}\newcommand{\bivarepsilon}{\boldsymbol{\varepsilon}}\newcommand{\bizeta}{\boldsymbol{\zeta}}\newcommand{\bieta}{\boldsymbol{\eta}}\newcommand{\bitheta}{\boldsymbol{\theta}}\newcommand{\biiota}{\boldsymbol{\iota}}\newcommand{\bikappa}{\boldsymbol{\kappa}}\newcommand{\bilambda}{\boldsymbol{\lambda}}\newcommand{\bimu}{\boldsymbol{\mu}}\newcommand{\binu}{\boldsymbol{\nu}}\newcommand{\bixi}{\boldsymbol{\xi}}\newcommand{\biomicron}{\boldsymbol{\micron}}\newcommand{\bipi}{\boldsymbol{\pi}}\newcommand{\birho}{\boldsymbol{\rho}}\newcommand{\bisigma}{\boldsymbol{\sigma}}\newcommand{\bitau}{\boldsymbol{\tau}}\newcommand{\biupsilon}{\boldsymbol{\upsilon}}\newcommand{\biphi}{\boldsymbol{\phi}}\newcommand{\bichi}{\boldsymbol{\chi}}\newcommand{\bipsi}{\boldsymbol{\psi}}\newcommand{\biomega}{\boldsymbol{\omega}}\begin{equation}\tag{7}p\left( {{B_l},t} \right) = 0 \quad {\rm{and}} \quad p \left( {{B_u},t} \right) = 0 \end{equation}

where $\newcommand{\bialpha}{\boldsymbol{\alpha}}\newcommand{\bibeta}{\boldsymbol{\beta}}\newcommand{\bigamma}{\boldsymbol{\gamma}}\newcommand{\bidelta}{\boldsymbol{\delta}}\newcommand{\bivarepsilon}{\boldsymbol{\varepsilon}}\newcommand{\bizeta}{\boldsymbol{\zeta}}\newcommand{\bieta}{\boldsymbol{\eta}}\newcommand{\bitheta}{\boldsymbol{\theta}}\newcommand{\biiota}{\boldsymbol{\iota}}\newcommand{\bikappa}{\boldsymbol{\kappa}}\newcommand{\bilambda}{\boldsymbol{\lambda}}\newcommand{\bimu}{\boldsymbol{\mu}}\newcommand{\binu}{\boldsymbol{\nu}}\newcommand{\bixi}{\boldsymbol{\xi}}\newcommand{\biomicron}{\boldsymbol{\micron}}\newcommand{\bipi}{\boldsymbol{\pi}}\newcommand{\birho}{\boldsymbol{\rho}}\newcommand{\bisigma}{\boldsymbol{\sigma}}\newcommand{\bitau}{\boldsymbol{\tau}}\newcommand{\biupsilon}{\boldsymbol{\upsilon}}\newcommand{\biphi}{\boldsymbol{\phi}}\newcommand{\bichi}{\boldsymbol{\chi}}\newcommand{\bipsi}{\boldsymbol{\psi}}\newcommand{\biomega}{\boldsymbol{\omega}}\delta \left( v \right)$ denotes a delta function, and $\newcommand{\bialpha}{\boldsymbol{\alpha}}\newcommand{\bibeta}{\boldsymbol{\beta}}\newcommand{\bigamma}{\boldsymbol{\gamma}}\newcommand{\bidelta}{\boldsymbol{\delta}}\newcommand{\bivarepsilon}{\boldsymbol{\varepsilon}}\newcommand{\bizeta}{\boldsymbol{\zeta}}\newcommand{\bieta}{\boldsymbol{\eta}}\newcommand{\bitheta}{\boldsymbol{\theta}}\newcommand{\biiota}{\boldsymbol{\iota}}\newcommand{\bikappa}{\boldsymbol{\kappa}}\newcommand{\bilambda}{\boldsymbol{\lambda}}\newcommand{\bimu}{\boldsymbol{\mu}}\newcommand{\binu}{\boldsymbol{\nu}}\newcommand{\bixi}{\boldsymbol{\xi}}\newcommand{\biomicron}{\boldsymbol{\micron}}\newcommand{\bipi}{\boldsymbol{\pi}}\newcommand{\birho}{\boldsymbol{\rho}}\newcommand{\bisigma}{\boldsymbol{\sigma}}\newcommand{\bitau}{\boldsymbol{\tau}}\newcommand{\biupsilon}{\boldsymbol{\upsilon}}\newcommand{\biphi}{\boldsymbol{\phi}}\newcommand{\bichi}{\boldsymbol{\chi}}\newcommand{\bipsi}{\boldsymbol{\psi}}\newcommand{\biomega}{\boldsymbol{\omega}}{B_l}$ and $\newcommand{\bialpha}{\boldsymbol{\alpha}}\newcommand{\bibeta}{\boldsymbol{\beta}}\newcommand{\bigamma}{\boldsymbol{\gamma}}\newcommand{\bidelta}{\boldsymbol{\delta}}\newcommand{\bivarepsilon}{\boldsymbol{\varepsilon}}\newcommand{\bizeta}{\boldsymbol{\zeta}}\newcommand{\bieta}{\boldsymbol{\eta}}\newcommand{\bitheta}{\boldsymbol{\theta}}\newcommand{\biiota}{\boldsymbol{\iota}}\newcommand{\bikappa}{\boldsymbol{\kappa}}\newcommand{\bilambda}{\boldsymbol{\lambda}}\newcommand{\bimu}{\boldsymbol{\mu}}\newcommand{\binu}{\boldsymbol{\nu}}\newcommand{\bixi}{\boldsymbol{\xi}}\newcommand{\biomicron}{\boldsymbol{\micron}}\newcommand{\bipi}{\boldsymbol{\pi}}\newcommand{\birho}{\boldsymbol{\rho}}\newcommand{\bisigma}{\boldsymbol{\sigma}}\newcommand{\bitau}{\boldsymbol{\tau}}\newcommand{\biupsilon}{\boldsymbol{\upsilon}}\newcommand{\biphi}{\boldsymbol{\phi}}\newcommand{\bichi}{\boldsymbol{\chi}}\newcommand{\bipsi}{\boldsymbol{\psi}}\newcommand{\biomega}{\boldsymbol{\omega}}{B_u}$ are, respectively, the lower and upper bounds. The first boundary condition (Equation 6) enforces that the diffusion process starts at zero and the second boundary condition (Equation 7) terminates the process as soon as one of the two decision bounds is reached. Response time distribution for each stimulus strength and choice was obtained by convolving the distribution of bound crossing times with the distribution of nondecision times. The distribution of nondecision times was a Gaussian, whose mean was a free model parameter ($\newcommand{\bialpha}{\boldsymbol{\alpha}}\newcommand{\bibeta}{\boldsymbol{\beta}}\newcommand{\bigamma}{\boldsymbol{\gamma}}\newcommand{\bidelta}{\boldsymbol{\delta}}\newcommand{\bivarepsilon}{\boldsymbol{\varepsilon}}\newcommand{\bizeta}{\boldsymbol{\zeta}}\newcommand{\bieta}{\boldsymbol{\eta}}\newcommand{\bitheta}{\boldsymbol{\theta}}\newcommand{\biiota}{\boldsymbol{\iota}}\newcommand{\bikappa}{\boldsymbol{\kappa}}\newcommand{\bilambda}{\boldsymbol{\lambda}}\newcommand{\bimu}{\boldsymbol{\mu}}\newcommand{\binu}{\boldsymbol{\nu}}\newcommand{\bixi}{\boldsymbol{\xi}}\newcommand{\biomicron}{\boldsymbol{\micron}}\newcommand{\bipi}{\boldsymbol{\pi}}\newcommand{\birho}{\boldsymbol{\rho}}\newcommand{\bisigma}{\boldsymbol{\sigma}}\newcommand{\bitau}{\boldsymbol{\tau}}\newcommand{\biupsilon}{\boldsymbol{\upsilon}}\newcommand{\biphi}{\boldsymbol{\phi}}\newcommand{\bichi}{\boldsymbol{\chi}}\newcommand{\bipsi}{\boldsymbol{\psi}}\newcommand{\biomega}{\boldsymbol{\omega}}\overline {T_0}$) and whose standard deviation was one third of the mean. Treating the standard deviation of nondecision time as an additional degree of freedom in the model did not change the results.

**Figure 4:**
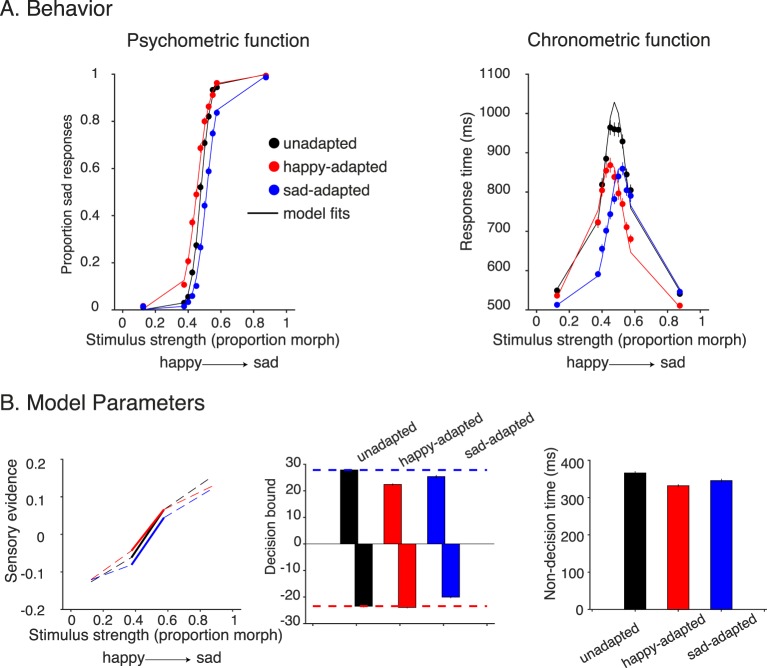
Adaptation results from Experiment 1 (fixed stimulus set). (A) Behavioral results of Experiment 1 from group data. The psychometric function (left) shows evidence of adaptation: Adapting happy or sad increased the likelihood of responding sad or happy, respectively. The chronometric functions (right) show a shift in the direction of adaptation, peaking at the PSE. In addition, adapting to happy or sad faces reduced the response time compared to no adaptation. Fitted curves on the psychometric and chronometric functions are derived from the same drift-diffusion model fits (one fit per adaptation condition). (B) Model parameters for the three adaptation conditions. Adaptation caused a shift in the sensory evidence plots (left) in the direction of adaptation. Adaptation also caused a reduction in decision bounds (middle), with a larger reduction for sad when adapting happy, and vice versa. Positive and negative bounds in the drift-diffusion model corresponded to sad and happy choices, respectively. Finally, there was a modest reduction in nondecision time during the adaptation conditions (right).

Because the numerical solution of Equation 5 is computationally intensive, we reduced the number of model parameters based on the results of our first fitting method, which showed linear change of drift rates for intermediate stimulus strengths (Figure 3C). Therefore, instead of separate drift rates for the 11 stimulus levels, we used four parameters: two parameters for the drift rates of the two extreme stimuli and two parameters to define a linear relationship between drift rates and stimulus strength for the middle nine stimuli (Figures 4B and 6B). This allowed us to reduce the number of free parameters from 14 to seven (two parameters for the bounds, one parameter for nondecision time, and four parameters for drift rates). The fits were performed independently for each of the three adaptation conditions. Adding more parameters to the model by assuming different nondecision times for the two choices (a total of eight parameters per condition) did not change the quality of the fits or our conclusions about changes of sensitivity and bound heights with adaptation (Supplementary Figures S2 and S3).

Standard errors of model parameters in both fitting methods were obtained by multiple fits to bootstrapped datasets (40–60 iterations) and calculating the standard deviation of model parameters across iterations.

#### Contribution analysis

Both changes in sensitivity (drift rates) and in decision bounds can alter the proportion of choices, and hence can contribute to a shift in the psychometric function during adaptation. For each adaptation condition, we quantified these contributions by dividing each experimentally observed shift into three components: (a) $\newcommand{\bialpha}{\boldsymbol{\alpha}}\newcommand{\bibeta}{\boldsymbol{\beta}}\newcommand{\bigamma}{\boldsymbol{\gamma}}\newcommand{\bidelta}{\boldsymbol{\delta}}\newcommand{\bivarepsilon}{\boldsymbol{\varepsilon}}\newcommand{\bizeta}{\boldsymbol{\zeta}}\newcommand{\bieta}{\boldsymbol{\eta}}\newcommand{\bitheta}{\boldsymbol{\theta}}\newcommand{\biiota}{\boldsymbol{\iota}}\newcommand{\bikappa}{\boldsymbol{\kappa}}\newcommand{\bilambda}{\boldsymbol{\lambda}}\newcommand{\bimu}{\boldsymbol{\mu}}\newcommand{\binu}{\boldsymbol{\nu}}\newcommand{\bixi}{\boldsymbol{\xi}}\newcommand{\biomicron}{\boldsymbol{\micron}}\newcommand{\bipi}{\boldsymbol{\pi}}\newcommand{\birho}{\boldsymbol{\rho}}\newcommand{\bisigma}{\boldsymbol{\sigma}}\newcommand{\bitau}{\boldsymbol{\tau}}\newcommand{\biupsilon}{\boldsymbol{\upsilon}}\newcommand{\biphi}{\boldsymbol{\phi}}\newcommand{\bichi}{\boldsymbol{\chi}}\newcommand{\bipsi}{\boldsymbol{\psi}}\newcommand{\biomega}{\boldsymbol{\omega}}{C_S}$, the expected shift of the psychometric function if bound heights remained the same as in the unadapted condition but drift rates changed by the amount in the model fit of the adapted condition, (b) $\newcommand{\bialpha}{\boldsymbol{\alpha}}\newcommand{\bibeta}{\boldsymbol{\beta}}\newcommand{\bigamma}{\boldsymbol{\gamma}}\newcommand{\bidelta}{\boldsymbol{\delta}}\newcommand{\bivarepsilon}{\boldsymbol{\varepsilon}}\newcommand{\bizeta}{\boldsymbol{\zeta}}\newcommand{\bieta}{\boldsymbol{\eta}}\newcommand{\bitheta}{\boldsymbol{\theta}}\newcommand{\biiota}{\boldsymbol{\iota}}\newcommand{\bikappa}{\boldsymbol{\kappa}}\newcommand{\bilambda}{\boldsymbol{\lambda}}\newcommand{\bimu}{\boldsymbol{\mu}}\newcommand{\binu}{\boldsymbol{\nu}}\newcommand{\bixi}{\boldsymbol{\xi}}\newcommand{\biomicron}{\boldsymbol{\micron}}\newcommand{\bipi}{\boldsymbol{\pi}}\newcommand{\birho}{\boldsymbol{\rho}}\newcommand{\bisigma}{\boldsymbol{\sigma}}\newcommand{\bitau}{\boldsymbol{\tau}}\newcommand{\biupsilon}{\boldsymbol{\upsilon}}\newcommand{\biphi}{\boldsymbol{\phi}}\newcommand{\bichi}{\boldsymbol{\chi}}\newcommand{\bipsi}{\boldsymbol{\psi}}\newcommand{\biomega}{\boldsymbol{\omega}}{C_B}$, the expected shift if drift rates remained the same as in the unadapted condition but decision bounds changed by the amount in the model fit of the adapted condition, and (c) $\newcommand{\bialpha}{\boldsymbol{\alpha}}\newcommand{\bibeta}{\boldsymbol{\beta}}\newcommand{\bigamma}{\boldsymbol{\gamma}}\newcommand{\bidelta}{\boldsymbol{\delta}}\newcommand{\bivarepsilon}{\boldsymbol{\varepsilon}}\newcommand{\bizeta}{\boldsymbol{\zeta}}\newcommand{\bieta}{\boldsymbol{\eta}}\newcommand{\bitheta}{\boldsymbol{\theta}}\newcommand{\biiota}{\boldsymbol{\iota}}\newcommand{\bikappa}{\boldsymbol{\kappa}}\newcommand{\bilambda}{\boldsymbol{\lambda}}\newcommand{\bimu}{\boldsymbol{\mu}}\newcommand{\binu}{\boldsymbol{\nu}}\newcommand{\bixi}{\boldsymbol{\xi}}\newcommand{\biomicron}{\boldsymbol{\micron}}\newcommand{\bipi}{\boldsymbol{\pi}}\newcommand{\birho}{\boldsymbol{\rho}}\newcommand{\bisigma}{\boldsymbol{\sigma}}\newcommand{\bitau}{\boldsymbol{\tau}}\newcommand{\biupsilon}{\boldsymbol{\upsilon}}\newcommand{\biphi}{\boldsymbol{\phi}}\newcommand{\bichi}{\boldsymbol{\chi}}\newcommand{\bipsi}{\boldsymbol{\psi}}\newcommand{\biomega}{\boldsymbol{\omega}}{C_{B \times S}}$, the expected shift from the interaction of changes in drift rates and bound heights. To calculate $\newcommand{\bialpha}{\boldsymbol{\alpha}}\newcommand{\bibeta}{\boldsymbol{\beta}}\newcommand{\bigamma}{\boldsymbol{\gamma}}\newcommand{\bidelta}{\boldsymbol{\delta}}\newcommand{\bivarepsilon}{\boldsymbol{\varepsilon}}\newcommand{\bizeta}{\boldsymbol{\zeta}}\newcommand{\bieta}{\boldsymbol{\eta}}\newcommand{\bitheta}{\boldsymbol{\theta}}\newcommand{\biiota}{\boldsymbol{\iota}}\newcommand{\bikappa}{\boldsymbol{\kappa}}\newcommand{\bilambda}{\boldsymbol{\lambda}}\newcommand{\bimu}{\boldsymbol{\mu}}\newcommand{\binu}{\boldsymbol{\nu}}\newcommand{\bixi}{\boldsymbol{\xi}}\newcommand{\biomicron}{\boldsymbol{\micron}}\newcommand{\bipi}{\boldsymbol{\pi}}\newcommand{\birho}{\boldsymbol{\rho}}\newcommand{\bisigma}{\boldsymbol{\sigma}}\newcommand{\bitau}{\boldsymbol{\tau}}\newcommand{\biupsilon}{\boldsymbol{\upsilon}}\newcommand{\biphi}{\boldsymbol{\phi}}\newcommand{\bichi}{\boldsymbol{\chi}}\newcommand{\bipsi}{\boldsymbol{\psi}}\newcommand{\biomega}{\boldsymbol{\omega}}{C_S}$ and $\newcommand{\bialpha}{\boldsymbol{\alpha}}\newcommand{\bibeta}{\boldsymbol{\beta}}\newcommand{\bigamma}{\boldsymbol{\gamma}}\newcommand{\bidelta}{\boldsymbol{\delta}}\newcommand{\bivarepsilon}{\boldsymbol{\varepsilon}}\newcommand{\bizeta}{\boldsymbol{\zeta}}\newcommand{\bieta}{\boldsymbol{\eta}}\newcommand{\bitheta}{\boldsymbol{\theta}}\newcommand{\biiota}{\boldsymbol{\iota}}\newcommand{\bikappa}{\boldsymbol{\kappa}}\newcommand{\bilambda}{\boldsymbol{\lambda}}\newcommand{\bimu}{\boldsymbol{\mu}}\newcommand{\binu}{\boldsymbol{\nu}}\newcommand{\bixi}{\boldsymbol{\xi}}\newcommand{\biomicron}{\boldsymbol{\micron}}\newcommand{\bipi}{\boldsymbol{\pi}}\newcommand{\birho}{\boldsymbol{\rho}}\newcommand{\bisigma}{\boldsymbol{\sigma}}\newcommand{\bitau}{\boldsymbol{\tau}}\newcommand{\biupsilon}{\boldsymbol{\upsilon}}\newcommand{\biphi}{\boldsymbol{\phi}}\newcommand{\bichi}{\boldsymbol{\chi}}\newcommand{\bipsi}{\boldsymbol{\psi}}\newcommand{\biomega}{\boldsymbol{\omega}}{C_B}$, we used the drift-diffusion model and the specified model parameters to generate predictions for the probabilities of sad and happy responses for each stimulus strength in the adaptation condition. We fit Equation 1 to these predicted probabilities to calculate the expected PSE and subtracted the unadapted PSE from it to get the expected shift. We then calculated the interaction effect ($\newcommand{\bialpha}{\boldsymbol{\alpha}}\newcommand{\bibeta}{\boldsymbol{\beta}}\newcommand{\bigamma}{\boldsymbol{\gamma}}\newcommand{\bidelta}{\boldsymbol{\delta}}\newcommand{\bivarepsilon}{\boldsymbol{\varepsilon}}\newcommand{\bizeta}{\boldsymbol{\zeta}}\newcommand{\bieta}{\boldsymbol{\eta}}\newcommand{\bitheta}{\boldsymbol{\theta}}\newcommand{\biiota}{\boldsymbol{\iota}}\newcommand{\bikappa}{\boldsymbol{\kappa}}\newcommand{\bilambda}{\boldsymbol{\lambda}}\newcommand{\bimu}{\boldsymbol{\mu}}\newcommand{\binu}{\boldsymbol{\nu}}\newcommand{\bixi}{\boldsymbol{\xi}}\newcommand{\biomicron}{\boldsymbol{\micron}}\newcommand{\bipi}{\boldsymbol{\pi}}\newcommand{\birho}{\boldsymbol{\rho}}\newcommand{\bisigma}{\boldsymbol{\sigma}}\newcommand{\bitau}{\boldsymbol{\tau}}\newcommand{\biupsilon}{\boldsymbol{\upsilon}}\newcommand{\biphi}{\boldsymbol{\phi}}\newcommand{\bichi}{\boldsymbol{\chi}}\newcommand{\bipsi}{\boldsymbol{\psi}}\newcommand{\biomega}{\boldsymbol{\omega}}{C_{B \times S}}$) as $\newcommand{\bialpha}{\boldsymbol{\alpha}}\newcommand{\bibeta}{\boldsymbol{\beta}}\newcommand{\bigamma}{\boldsymbol{\gamma}}\newcommand{\bidelta}{\boldsymbol{\delta}}\newcommand{\bivarepsilon}{\boldsymbol{\varepsilon}}\newcommand{\bizeta}{\boldsymbol{\zeta}}\newcommand{\bieta}{\boldsymbol{\eta}}\newcommand{\bitheta}{\boldsymbol{\theta}}\newcommand{\biiota}{\boldsymbol{\iota}}\newcommand{\bikappa}{\boldsymbol{\kappa}}\newcommand{\bilambda}{\boldsymbol{\lambda}}\newcommand{\bimu}{\boldsymbol{\mu}}\newcommand{\binu}{\boldsymbol{\nu}}\newcommand{\bixi}{\boldsymbol{\xi}}\newcommand{\biomicron}{\boldsymbol{\micron}}\newcommand{\bipi}{\boldsymbol{\pi}}\newcommand{\birho}{\boldsymbol{\rho}}\newcommand{\bisigma}{\boldsymbol{\sigma}}\newcommand{\bitau}{\boldsymbol{\tau}}\newcommand{\biupsilon}{\boldsymbol{\upsilon}}\newcommand{\biphi}{\boldsymbol{\phi}}\newcommand{\bichi}{\boldsymbol{\chi}}\newcommand{\bipsi}{\boldsymbol{\psi}}\newcommand{\biomega}{\boldsymbol{\omega}}{\it{total\ shift}} - {C_S} - {C_B}$. To calculate the overall contribution of the changes of bound heights and sensitivity to the observed shifts of the psychometric functions, we evenly divided the shift due to the interaction of parameters. That is, the overall contribution of changes of bound heights equals $\newcommand{\bialpha}{\boldsymbol{\alpha}}\newcommand{\bibeta}{\boldsymbol{\beta}}\newcommand{\bigamma}{\boldsymbol{\gamma}}\newcommand{\bidelta}{\boldsymbol{\delta}}\newcommand{\bivarepsilon}{\boldsymbol{\varepsilon}}\newcommand{\bizeta}{\boldsymbol{\zeta}}\newcommand{\bieta}{\boldsymbol{\eta}}\newcommand{\bitheta}{\boldsymbol{\theta}}\newcommand{\biiota}{\boldsymbol{\iota}}\newcommand{\bikappa}{\boldsymbol{\kappa}}\newcommand{\bilambda}{\boldsymbol{\lambda}}\newcommand{\bimu}{\boldsymbol{\mu}}\newcommand{\binu}{\boldsymbol{\nu}}\newcommand{\bixi}{\boldsymbol{\xi}}\newcommand{\biomicron}{\boldsymbol{\micron}}\newcommand{\bipi}{\boldsymbol{\pi}}\newcommand{\birho}{\boldsymbol{\rho}}\newcommand{\bisigma}{\boldsymbol{\sigma}}\newcommand{\bitau}{\boldsymbol{\tau}}\newcommand{\biupsilon}{\boldsymbol{\upsilon}}\newcommand{\biphi}{\boldsymbol{\phi}}\newcommand{\bichi}{\boldsymbol{\chi}}\newcommand{\bipsi}{\boldsymbol{\psi}}\newcommand{\biomega}{\boldsymbol{\omega}}{C_B} + 0.5{C_{B \times S}}$, and the overall contribution of changes of sensitivity equals $\newcommand{\bialpha}{\boldsymbol{\alpha}}\newcommand{\bibeta}{\boldsymbol{\beta}}\newcommand{\bigamma}{\boldsymbol{\gamma}}\newcommand{\bidelta}{\boldsymbol{\delta}}\newcommand{\bivarepsilon}{\boldsymbol{\varepsilon}}\newcommand{\bizeta}{\boldsymbol{\zeta}}\newcommand{\bieta}{\boldsymbol{\eta}}\newcommand{\bitheta}{\boldsymbol{\theta}}\newcommand{\biiota}{\boldsymbol{\iota}}\newcommand{\bikappa}{\boldsymbol{\kappa}}\newcommand{\bilambda}{\boldsymbol{\lambda}}\newcommand{\bimu}{\boldsymbol{\mu}}\newcommand{\binu}{\boldsymbol{\nu}}\newcommand{\bixi}{\boldsymbol{\xi}}\newcommand{\biomicron}{\boldsymbol{\micron}}\newcommand{\bipi}{\boldsymbol{\pi}}\newcommand{\birho}{\boldsymbol{\rho}}\newcommand{\bisigma}{\boldsymbol{\sigma}}\newcommand{\bitau}{\boldsymbol{\tau}}\newcommand{\biupsilon}{\boldsymbol{\upsilon}}\newcommand{\biphi}{\boldsymbol{\phi}}\newcommand{\bichi}{\boldsymbol{\chi}}\newcommand{\bipsi}{\boldsymbol{\psi}}\newcommand{\biomega}{\boldsymbol{\omega}}{C_S} + 0.5{C_{B \times S}}$.

## Results

### Validity of the stimulus set

Our stimulus set comprised equally spaced points along a morph trajectory. However, perceptual spaces and physical spaces need not correspond, so the perceptual strength of the stimuli must be determined empirically. We assessed the relationship between the physical and perceptual spaces in the no-adaptation condition in three ways. First, we examined the psychometric functions, expressed as the percentage of sad judgments versus stimulus strength. For each observer, the psychometric functions were approximately monotonic and well fit by a logistic function, in both Experiments 1 and 2 (Figure 3A). This indicates that the stimulus strength according to the morph trajectory monotonically matched perceptual judgments: The closer a stimulus was to the happy or sad end point, the more likely it was to be judged happy or sad, respectively. Moreover, the psychometric functions were highly similar for the two experiments in most observers, indicating that the slight difference in experimental design (choosing the range of stimuli based on practice blocks) did not substantially alter baseline performance.

Second, we made a more quantitative assessment of the perceptual spacing by comparing the stimulus strength with the log-odds (logit) of the judgments. Although the logit and the probabilities are simple monotonic transforms, the logit fit is easier to assess by visual inspection: According to a simple model of perceptual decision making via diffusion to fixed and symmetric bounds, the logit of the probability of judgments is a linear function of sensory evidence available for the choice (Kiani et al., 2014; Link, 1992; Palmer et al., 2005). For the nine intermediate stimuli tested, the logit changed linearly with stimulus strength (Figure 3B). The linear relationship did not hold for the two extreme stimulus values tested. This could either be because the perceptual space was warped at extreme values (relative to the physical space), or because we could not accurately measure the logit when the probabilities were close to 0 or 1.

Finally, we fit the judgments and response times with a drift-diffusion model, allowing for asymmetric bounds, and asked whether the fitted parameters for sensory evidence (drift rates) scaled with the physical stimulus values. We used the first fitting strategy described in the Methods (Equations 3 and 4), in which there was a free parameter for the drift rate of each stimulus strength. This test, unlike the psychometric functions, makes use of both response time and judgments. As with the logit of the judgments alone, the parameters for sensory evidence (drift rate) were close to linear for intermediate stimulus values, but not the two extreme values (Figure 3C). These results together indicate that the perceptual space is approximately linear with respect to the physical space over a substantial range, justifying the reduction in the number of free parameters for subsequent model fitting.

### Experiment 1: Adaptation to facial expression affects both sensitivity and decision bounds

For Experiment 1, the test stimuli were identical across adaptation conditions and participants. As expected from prior literature (Hsu & Young, 2004; Russell & Fehr, 1987; Webster et al., 2004; Xu et al., 2008), adapting to a facial expression caused the psychometric function to shift toward the adapting stimulus (Figure 4A, left). This means that adaptation to a sad expression made participants more likely to judge the test stimulus as happy, and adaptation to a happy expression made participants more likely to judge the test stimulus as sad. The PSE shifted by −0.028 ± 0.002 (pooled data, *p* < 10^−8^; median shift for individual subjects, −0.022; for five out of 6 subjects *p* ≤ 0.05) and 0.039 ± 0.002 (pooled data, *p* < 10^−8^; median shift for individual subjects, 0.022; for all subjects *p* ≤ 0.05) in the expected directions during adaptation to the happy and sad images, respectively. The difference in the PSE between the two adapt conditions (0.067) corresponds to approximately 2.7 steps in the 41-image morph line (Supplementary Figure S1).

Adaptation also altered the chronometric functions. An unexpected result was that relative to no adaptation, adapting to a happy or sad expression substantially reduced the overall response time: Participants responded about 100 ms faster to stimuli at or near the PSEs following adaptation to either facial expression compared to no adaptation (Figure 4A, right). Furthermore, the peak of the response time distributions shifted toward the adaptor, so that the most ambiguous stimulus (the stimulus closest to the PSE in each condition) had the slowest response times.

The full data, including the psychometric and chronometric functions, were fit by the drift-diffusion model applied to individual trial data (second fitting strategy in methods, Equations 5 through 7). The diffusion model overall did a good job of simultaneously fitting the psychometric and chronometric functions (Figure 4A), indicating that across our stimulus set, task, and adaptation conditions, performance can be accurately described by a simple drift-diffusion model of decision making. This is compatible with integration of sensory evidence for face discrimination as shown before (Okazawa et al., 2016). The advantage of the model fit is that it allows us to ask what aspects of the decision-making process explain the pattern of behavioral results.

**Figure 5:**
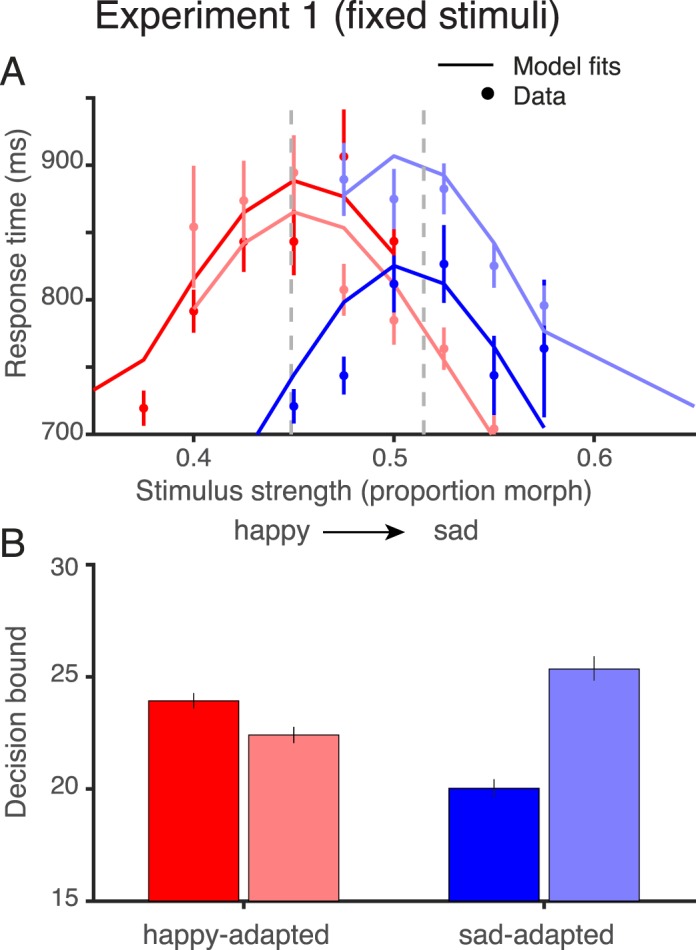
Chronometric functions by response for Experiment 1. (A) Response times as a function of stimulus strength (x-axis), adaptation condition (happy in red, sad in blue), and choice (happy responses with darker colors; sad responses with lighter colors). The stimulus range is reduced to emphasize the data near the PSEs. The PSEs are indicated by the dashed vertical lines. Lines are model fits. (B) The absolute value of the decision bounds, with darker colors for the happy bound and lighter colors for the sad bounds, replotted from Figure 4. An asymmetry in the response time for stimuli near the PSE (upper panel) is reflected in an asymmetry in the decision bound (lower panel).

**Figure 6:**
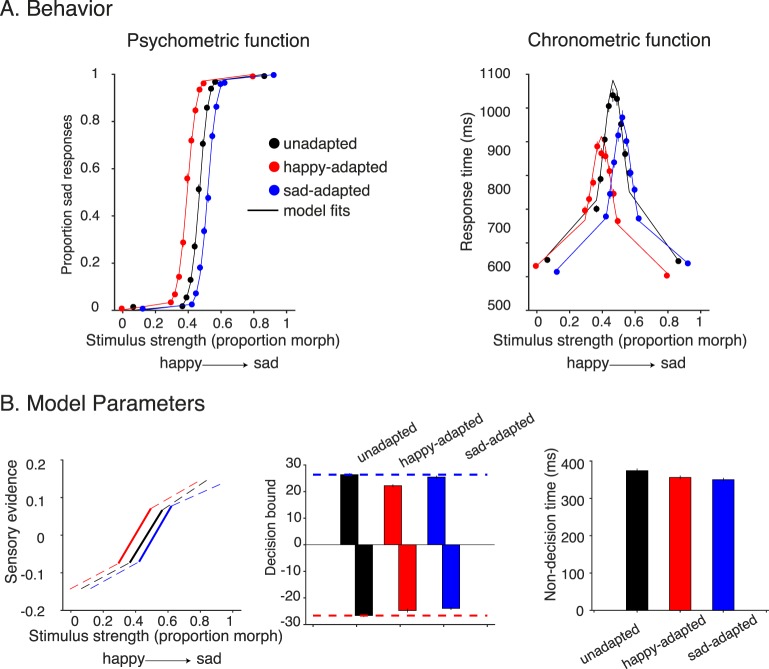
Adaptation results from Experiment 2 (balanced responses). Same as Figure 4, but for Experiment 2 in which the stimulus set was adjusted to ensure that responses were balanced in each adaptation condition.

**Figure 7:**
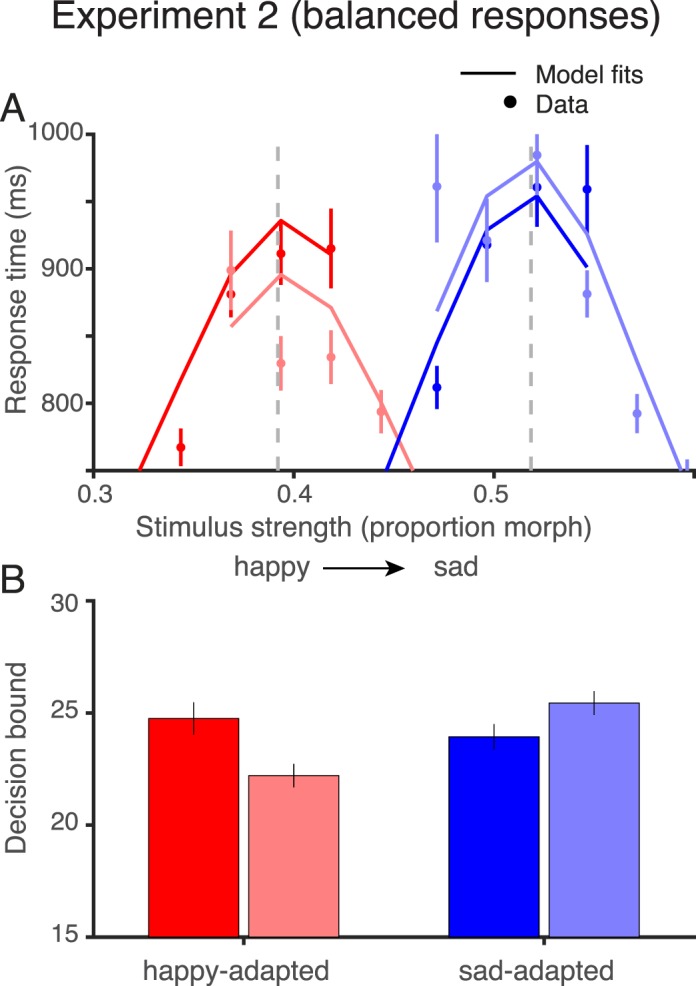
Chronometric functions by response for Experiment 2. Same as Figure 5, but for Experiment 2. When participants adapted to the happy face (red plots) the response time was slower for happy judgments than sad judgments (upper panel), consistent with a lower bound for sad faces (lower panel). When adapting sad, the pattern was reversed, but the effect was much smaller.

The change in performance following adaptation was primarily explained by three features of the diffusion model. First, the drift rate (or sensitivity) of each stimulus shifted toward the adaptor (Figure 4B). This observation is consistent with a long history of studies suggesting that adaptation causes a change in sensory representation. For example, a stimulus that was considered neutral in the unadapted experiment (drift rate 0) was interpreted as providing evidence for a sad decision (positive drift rate) following adaptation to a happy face.

Second, and more surprisingly, the decision bound was systematically lower during adaptation. Treating all bounds as positive, the average of the two bounds was lower following adaptation to either the happy or sad expression, compared to no adaptation (Figure 4B, middle). The decrease in bound height was 9.6% and 11.5% for the happy-adapted and sad-adapted trials compared to no adaptation (*z* test on model parameters, pooled data, *p* < 10^−8^; median bound reduction for individual subjects, 7.5% for happy-adapted and 10.8% for sad-adapted; for sad-adapted, three subjects *p* ≤ 0.05; for happy-adapted, two subjects *p* < 0.05; combined changes across subjects, bootstrap *p* < 10^−4^). This inference from model parameters was clearly reflected in the overall reduction in response time during adaptation (Figure 4A, upper right). A reduction in bounds indicates that participants made their judgments based on the accumulation of less data, and therefore predicts that under adaptation, participants should be less accurate. This is indeed what we observed, in that the psychometric functions became 20.1% shallower during adaptation (pooled data, $\newcommand{\bialpha}{\boldsymbol{\alpha}}\newcommand{\bibeta}{\boldsymbol{\beta}}\newcommand{\bigamma}{\boldsymbol{\gamma}}\newcommand{\bidelta}{\boldsymbol{\delta}}\newcommand{\bivarepsilon}{\boldsymbol{\varepsilon}}\newcommand{\bizeta}{\boldsymbol{\zeta}}\newcommand{\bieta}{\boldsymbol{\eta}}\newcommand{\bitheta}{\boldsymbol{\theta}}\newcommand{\biiota}{\boldsymbol{\iota}}\newcommand{\bikappa}{\boldsymbol{\kappa}}\newcommand{\bilambda}{\boldsymbol{\lambda}}\newcommand{\bimu}{\boldsymbol{\mu}}\newcommand{\binu}{\boldsymbol{\nu}}\newcommand{\bixi}{\boldsymbol{\xi}}\newcommand{\biomicron}{\boldsymbol{\micron}}\newcommand{\bipi}{\boldsymbol{\pi}}\newcommand{\birho}{\boldsymbol{\rho}}\newcommand{\bisigma}{\boldsymbol{\sigma}}\newcommand{\bitau}{\boldsymbol{\tau}}\newcommand{\biupsilon}{\boldsymbol{\upsilon}}\newcommand{\biphi}{\boldsymbol{\phi}}\newcommand{\bichi}{\boldsymbol{\chi}}\newcommand{\bipsi}{\boldsymbol{\psi}}\newcommand{\biomega}{\boldsymbol{\omega}}{\beta _4}$= −6.24 ± 0.90, *p* < 10^−8^, Equation 2).

Finally, we observed that the decision bounds became asymmetric during adaptation. In particular, the happy-adapted condition caused a lower bound for sad judgments (pooled data, 22.4 ± 0.4 vs. 27.8 ± 0.4, *p* < 10^−8^; median bound reduction for individual subjects, 10.1%; the reduction was significant for three out of six subjects with *p* ≤ 0.05; combined changes across subjects, bootstrap *p* < 10^−4^), and the sad-adapted condition caused a lower bound for happy judgments (20.0 ± 0.4 vs. 23.4 ± 0.4, *p* < 10^−8^; median bound reduction for individual subjects, 10.0%; the reduction was significant for three subjects with *p* ≤ 0.05; combined changes across subjects, bootstrap *p* < 10^−4^). A prediction made by the diffusion model we employed is that if the bounds for the two decisions are asymmetric, then the distribution of response times will differ for the two decisions, even for a fixed stimulus: Specifically, the response times for the decision with the lower bound will be shorter. This prediction was largely borne out by the data (Figure 5). In the sad-adapted condition, happy responses were about 100 ms faster than sad responses for stimuli near the PSE (Figure 5A). Because the figure zooms in on the response times for the ambiguous stimuli, it amplifies apparent discrepancies between the model and data. Better fits can be achieved by adding an urgency function to the model (Churchland, Kiani, & Shadlen, 2008; Hanks, Kiani, & Shadlen, 2014). However, because the discrepancies between model and data tend to be small (<50 ms), we opted for a simpler model without urgency to lower the number of model parameters. The faster happy responses for the sad-adapted condition explains why the model fit the boundaries asymmetrically, with a lower bound for happy decisions when adapting to sad faces (Figure 5B). The effect was similar, though smaller in magnitude and noisier, for the happy-adapted condition.

Importantly, an asymmetry in decision bounds makes one response more likely than the other, and hence can contribute to a shift in the psychometric function. The decision-bound effects show that a change in the decision-making process can complement changes in sensory representation during adaptation, with both effects contributing to the usual hallmark of aftereffects from adaptation: a shift in the psychometric function toward the adapting stimulus.

### Experiment 2: Adaptation with balanced responses

The first experiment showed that adaptation resulted in changes in both sensory representations and in the decision-making process. Because we used a method of constant stimuli, and because adaptation caused the PSE to shift toward the adapting stimulus, adaptation also caused an imbalance in the proportion of responses. For example, Participant 1's percentage of sad responses increased from 49% (unadapted) to 70% (happy-adapted) in Experiment 1. One possibility is that a bias in the response frequency caused participants to lower their decision bound for the more frequent response. Such a possibility is supported by studies showing that increased target frequency tends to result in subjects lowering their decision bound to respond more frequently to that target (Hanks, Mazurek, Kiani, Hopp, & Shadlen, 2011; Link, 1992; Mulder, Wagenmakers, Ratcliff, Boekel, & Forstmann, 2012). This process might have occurred in Experiment 1, such that an initial change in sensory representation following adaptation resulted in an increased frequency of making a particular response, and the increased frequency of this response then resulted in a lowering of the decision bound to make this response. If this were the explanation for the change in bounds observed in Experiment 1, then we would predict that when responses were balanced, there would be little or no change in decision bounds from adaptation.

To examine the effects of adaptation when responses were balanced, we designed Experiment 2 such that the proportion of responses was close to 50% for all participants and all adaptation conditions. Several features of the responses replicated Experiment 1, but there were important differences as well.

First, as in Experiment 1, adaptation caused a reliable shift in the psychometric function toward the adapting stimulus (Figure 6A, left; PSE shift for happy-adapted condition, pooled data, −0.073 ± 0.002, *p* < 10^−8^; median for individual subjects, −0.071; for all subjects *p* ≤ 0.05; PSE shift for sad-adapted condition, pooled data 0.054 ± 0.002, *p* < 10^−8^; median for individual subjects, 0.051; for all subjects *p* ≤ 0.05). Second, as in Experiment 1, adaptation caused a reduction in response time (Figure 6A, right), which the diffusion model attributed to a reduction of decision bound with adaptation (a reduction of 11.4% for the happy-adapted condition, *p* = 2.2 × 10^−7^, and 6.8% for the sad-adapted condition, *p* = 8.5 × 10^−4^, compared to the unadapted condition; single subject results: happy-adapted condition, median 12.2%, for five out of six subjects *p* ≤ 0.05, bootstrap *p* < 10^−4^ for combined changes across subjects; sad-adapted condition, median 10.1%, for four subjects *p* ≤ 0.05, bootstrap *p* < 10^−4^ combined changes across subjects). This reduction in decision bound was slightly less pronounced than in Experiment 1, and unlike Experiment 1, there was little flattening of the psychometric function ($\newcommand{\bialpha}{\boldsymbol{\alpha}}\newcommand{\bibeta}{\boldsymbol{\beta}}\newcommand{\bigamma}{\boldsymbol{\gamma}}\newcommand{\bidelta}{\boldsymbol{\delta}}\newcommand{\bivarepsilon}{\boldsymbol{\varepsilon}}\newcommand{\bizeta}{\boldsymbol{\zeta}}\newcommand{\bieta}{\boldsymbol{\eta}}\newcommand{\bitheta}{\boldsymbol{\theta}}\newcommand{\biiota}{\boldsymbol{\iota}}\newcommand{\bikappa}{\boldsymbol{\kappa}}\newcommand{\bilambda}{\boldsymbol{\lambda}}\newcommand{\bimu}{\boldsymbol{\mu}}\newcommand{\binu}{\boldsymbol{\nu}}\newcommand{\bixi}{\boldsymbol{\xi}}\newcommand{\biomicron}{\boldsymbol{\micron}}\newcommand{\bipi}{\boldsymbol{\pi}}\newcommand{\birho}{\boldsymbol{\rho}}\newcommand{\bisigma}{\boldsymbol{\sigma}}\newcommand{\bitau}{\boldsymbol{\tau}}\newcommand{\biupsilon}{\boldsymbol{\upsilon}}\newcommand{\biphi}{\boldsymbol{\phi}}\newcommand{\bichi}{\boldsymbol{\chi}}\newcommand{\bipsi}{\boldsymbol{\psi}}\newcommand{\biomega}{\boldsymbol{\omega}}{\beta _4}$ = −0.41 ± 1.0, *p* = 0.68, Equation 2). Furthermore, there was an asymmetry in the decision bounds following adaptation (Figure 7). We observed a lower bound for sad judgments in the happy-adapted condition (pooled data, 22.2 ± 0.5 vs. 26.4 ± 0.5, *p* < 10^−8^; median bound reduction for individual subjects, 12.8%; the reduction was significant for five out of six subjects with *p* ≤ 0.05; combined changes across subjects, bootstrap *p* < 10^−4^), and a lower bound for happy judgments in the sad-adapted condition (23.9 ± 0.6 vs. 26.6 ± 0.6, *p* = 1.7 × 10^−6^; median bound reduction for individual subjects, 10.3%; the reduction was significant for four subjects with *p* ≤ 0.05; combined changes across subjects, bootstrap *p* < 10^−4^).

The response time asymmetry was a less pronounced effect compared to Experiment 1. In contrast, the change in representation, indicated by the shift in drift rates, was larger for Experiment 2 than Experiment 1. Together these results suggest that when the test stimuli are arranged such that the participant's responses are approximately balanced, adaptation has a larger effect on sensory representations and a smaller effect on decision strategy. We compare these phenomena quantitatively in the next section.

### Contributions of sensitivity and decision bounds to shifts in psychometric functions

**Figure 8:**
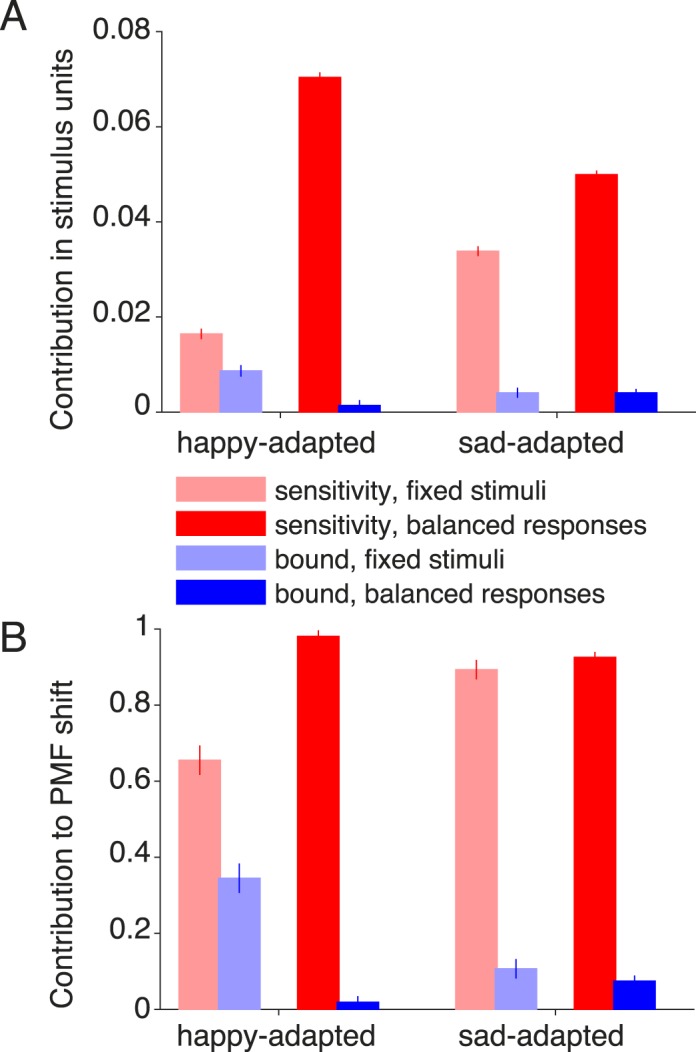
Contribution of model parameters to shift in psychometric function. (A) The shifts in the psychometric functions resulting from adaptation were partitioned into the contributions from sensitivity changes and from bound changes. Each bar represents the contribution to the shift in the psychometric function attributed to one of the two types of parameters. Positive values indicate that the contribution was a shift in the direction toward the adapting stimulus. The sum of the two bars is equal to the amount that the PSE shifted, in units of stimulus strength, during happy-adapted or sad-adapted conditions compared to the unadapted condition. (B) Same as Panel A, but normalized to the total shift in the PSE so that each pair of bars sums to 1.

Both changes in sensory representation and changes in decision bounds arising from adaptation can cause a shift in the psychometric function. We parcellated the shift in each of four conditions (happy-adapted and sad-adapted conditions from Experiments 1 and 2) into the separate contributions from the change in sensory representation (drift rates) and change in decision bound (Figure 8). The analysis shows several clear patterns. First, in both experiments, the larger contribution to the PSE shift came from the change in sensitivity (red bars). Yet there was also a systematic effect of the decision bound on the shift of psychometric functions (blue bars), and this effect was larger in Experiment 1 than Experiment 2, both for happy- and sad-adapted conditions. Hence, when responses were balanced by choosing appropriate stimulus sets for each participant and each condition, adaptation was more strongly driven by a change in sensory representation and less strongly by a change in decision-making strategy. This pattern holds whether quantifying the contributions in units of stimulus strength (Figure 8A) or as a fraction of the shift in the PSE (Figure 8B). Note that this conclusion concerns the effects of drift rates and bound heights on shifts in the psychometric function and is independent of the separate finding that the overall bound heights declined in both experiments. Finally, the contributions from sensitivity and bound changes were in the same direction (i.e., all bars are positive). Had the bound changes and sensitivity changes pushed the psychometric function in opposite directions, then one of the two factors would have had a negative contribution. This indicates that both types of changes tended to push the psychometric function toward the adaptor.

## Discussion

Biological sensory systems are faced with the competing challenges of operating under an enormous range of possible stimulation levels while still maintaining the ability to respond to small changes in the input (Dunn, Lankheet, & Rieke, 2007). Given the limited resources that the nervous system has at its disposal, adapting to the observed or expected range of stimulation can be an important tool for meeting these dual demands (Rushton, 1965; Webster, 2015). A widely observed behavioral signature of adaptation is a shift in the perceived neutral point toward the adapting stimulus. The shift in the neutral point can be measured with various psychophysical tools such as the method of adjustment (Gibson, 1937; Gibson & Radner, 1937), a staircase (Webster et al., 2004), or by fitting a psychometric function using the method of constant stimuli (Blake & Hiris, 1993). We focus here on shifts in psychometric functions, but the same principles hold for other methods of measuring neutral points. While much past research on adaptation and other contextual effects have focused on the phenomenon of a shifted psychometric function (Afraz & Cavanagh, 2008; Blake & Hiris, 1993; Leopold, O'Toole, Vetter, & Blanz, 2001; Winawer, Huk, & Boroditsky, 2008, 2010), it provides neither a complete characterization of adaptation nor a mechanistically unambiguous description. Below, we explain the ambiguous nature of psychometric function shifts and use our response time experimental design to offer a way to parcellate the effects of sensory and decision-making mechanisms to this shift. We then expand our perspective to offer a broader and more complete description of adaptation as a normative process.

### Multiple factors can cause a shift in the psychometric function

**Figure 9:**
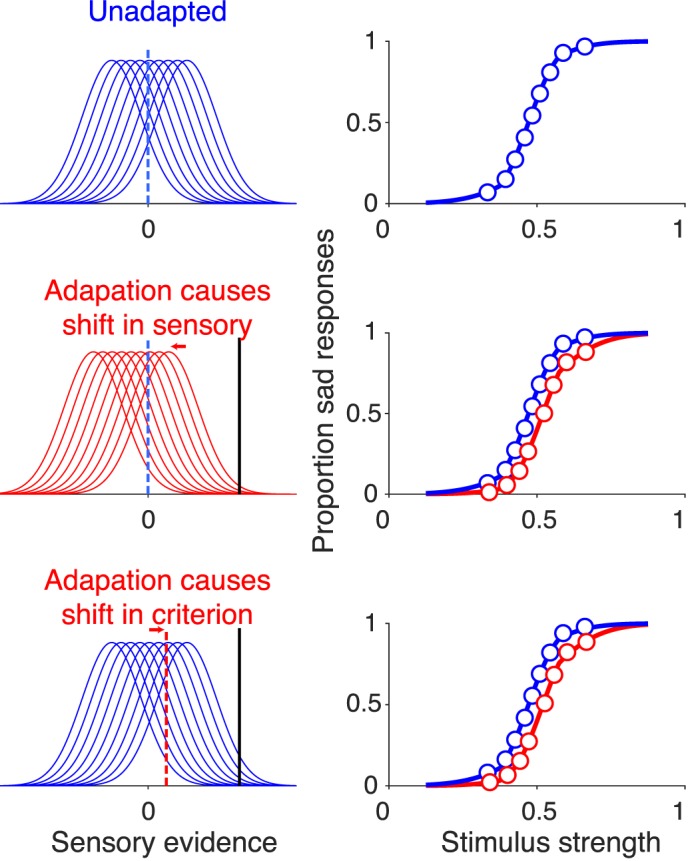
Adaptation from the perspective of signal detection theory. (Upper) The left panel shows hypothetical distributions of sensory evidence for nine stimulus levels in the unadapted condition. The stimuli can be thought of as the nine central stimuli in our first experiment, ranging from happy (leftmost curve) to sad (rightmost curve). The vertical dashed line is the criterion, assumed to be unbiased. On any given trial, a sensory response greater than the criterion results in a sad judgment, and lower than the criterion to a happy judgment. The proportion of sad judgments is plotted on the right, where the nine symbols correspond to the nine stimuli on the left. (Middle) Same as upper panel, but assuming that the participant has adapted to a sad stimulus (black vertical line), causing a leftward shift in sensory evidence, indicated by the red arrow and the shifted distributions, and no change in criterion. The shift in sensory evidence away from the adaptor translates to a psychometric function shifted toward the adaptor, shown as the red psychometric function on the right. (The blue psychometric function is replotted from the upper panel for comparison). (Lower) The third row is the same as the middle row, except that adaptation is assumed to shift the criterion (red dashed line) toward the adaptor, rather than shifting the internal responses away from the adaptor. This predicts a shift in the psychometric function that is identical to the shift in the middle panel.

As expected, we found a clear shift in the psychometric function in both of the experiments in this study. In principle, this type of shift can arise for multiple reasons. In the framework of signal detection theory, two equally valid explanations are a change in criterion (bias) and a change in the distributions of internal responses (representation; see Figure 9). The tools of signal detection theory allow one to infer the relative position of the criterion to the internal responses, but not the absolute quantities; hence, without a direct measure of the internal state, one cannot distinguish the two possibilities (Macmillan & Creelman, 2005). Both explanations are plausible, evidenced by past research. For example, with neural measurements, one can show unambiguous sensory shifts as a result of adaptation (Benucci, Saleem, & Carandini, 2013; Kohn & Movshon, 2004; Snow, Coen-Cagli, & Schwartz, 2016). And in behavioral experiments, explicit instruction to make biased guesses when uncertain (Morgan, Dillenburger, Raphael, & Solomon, 2012), or implicit cues by manipulation of prior probabilities (Hanks et al., 2011; Mulder et al., 2012; Rao, DeAngelis, & Snyder, 2012) or expected rewards (Rorie, Gao, McClelland, & Newsome, 2010), can cause shifts in the psychometric function.

Because of this ambiguity, when seeking to disentangle decisional and representational contributions to adaptation-related aftereffects, researchers have resorted to different, and often complicated, experimental paradigms designed to minimize the contribution of decisional biases (Morgan, 2013, 2014; Morgan et al., 2012). In these studies, the logic is that if an adaptation effect is observed in a design in which a response bias is unlikely to affect choice, then any measured aftereffect is a genuine perceptual effect rather than a bias. In our approach, rather than aiming for the absence of a decision-making bias, we sought to measure it. If decision-level biases are a fundamental part of sensory decision making, and are often a component of adaptation effects, as proposed recently by Mather and Sharman (2015), then it is important to be able to measure both effects in a single type of experiment and quantify their relative contributions.

### Response time provides the means for parcellating decisional and representational effects

Using established models of the decision-making process (Donkin et al., 2011; Link, 1992; Moran, 2015; Ratcliff & McKoon, 2008; Shadlen & Kiani, 2013), we proposed that response time provides leverage to parcellate effects of changes in sensory representation and changes in how the representation is used in making decisions. The results of our expression adaptation task show that both sensory and decision-making changes make significant contributions to the shift of the psychometric function. Further, changes of sensory representations are a dominant factor and changes of the decision-making process are modulated with the magnitude of response imbalance. The two effects are synergistic, in that they both tend to shift the psychometric function toward the adapting stimulus, resulting in more judgments opposite to the adapting stimulus (Figure 8). Our method, which can be applied to several existing adaptation paradigms and expanded to other tasks (see below), builds on the observation that changes in the decision bounds lead to distinct changes in response time distributions. One of the manifestations of these changes is an asymmetry in the chronometric function: Response time will be shorter when making one response compared to another even for the same stimulus (Figures 5 and 7). In contrast, a pure representational effect will modify the sensory evidence furnished by the test stimulus, and is expected to cause equivalent shifts in the subject's psychometric and chronometric functions (Hanks et al., 2011; Purcell & Kiani, 2016b). In any given experiment, a mixture of these effects might be observed. A model is required to quantitatively split the behavioral results into these different causal factors. By applying a drift-diffusion model to the results of a standard adaptation paradigm, we have quantified the relative contributions of bound and sensitivity in the same adaptation experiment.

In addition to changes of sensitivity and decision bound that influenced participants' choice and decision times, we also observed a small but consistent reduction of nondecision time in the adapted conditions compared to the unadapted condition (Figures 4B and Supplementary Figure S2B; also see Figures 6B and Supplementary Figure S3B). These reductions of nondecision time contributed to the overall decrease of response times in the adapted conditions. However, we cannot pinpoint the origin of this reduction. It could have been caused by adaptation or by higher predictability of the test stimulus onset in the adapted blocks. To equalize the interval between consecutive trials across conditions, we elongated the blank interval between trials in the unadapted condition by the duration of the top-up adapting stimuli in the adapted conditions (3.5 s total interval). However, this manipulation could have increased temporal uncertainty of the test stimulus onset, causing longer nondecision times in the unadapted condition. Because changes of nondecision time do not bear on the shift of the psychometric function or asymmetries in response times for the two choices, we focus our attention on changes of sensitivity and decision bounds with adaptation.

Several previous studies have reported changes of response time following adaptation. However, they typically did not distinguish between changes in nondecision time and decision time, or changes in sensory or decision-making processes that could modify the decision time. Some of these studies reported an elongation of response times (Hovland, 1936; Shulman, Sullivan, Gish, & Sakoda, 1986) and some a shortening of response times (Kompaniez-Dunigan, Abbey, Boone, & Webster, 2015; McDermott, Malkoc, Mulligan, & Webster, 2010; Wissig, Patterson, & Kohn, 2013). Many of these studies also showed a stimulus-dependent change in response times, with faster responses to stimuli that differ from the adaptor compared to stimuli similar to the adaptor (e.g., Hovland, 1936; Kompaniez-Dunigan et al., 2015; Mathot & Theeuwes, 2013). A stimulus-independent change is likely to be due to changes of nondecision time, but stimulus-dependent changes could be caused by both changes of sensitivity and changes of decision bounds. In light of our results, it will be fruitful to revisit those datasets and make attempts to dissect different factors that could have contributed to observed changes of response times. Such an exploration will generate new insights about the diversity of post-adaptation behavior and its potential causes.

### Adaptation as an integrated sensory and decision-making phenomenon

The insight that adaptation could be shaped by both sensory and decision-making processes leads to a new framework for understanding the neural mechanisms that underlie adaptation. An immediate insight in this framework is that the shift of the psychometric function is only one of the manifestations of adaptation, not a complete recapitulation of the phenomenon. An equally important manifestation is the change in response times, which show a multitude of alterations: shift (Figures 4 and 6), asymmetry (Figures 5 and 7), and an overall reduction (Figures 4 and 6). The reduction of the decision times indicates a decrease in bound heights and, thereby, accumulation of less sensory evidence for both choices in the adapted condition. This overall reduction of the accumulated evidence is a large effect (∼100 ms or 20% of the dynamic range of response times in the two experiments).

We speculate this reduction in response time has a normative basis. If the nervous system uses adaptation to adjust the range of stimuli it is most sensitive to (Kohn, 2007; Rieke & Rudd, 2009; Webster, 2015), then adaptation should have two mechanistic effects: limiting the range of sensory stimuli that the system expects to respond to and increasing the expected chance of a successful choice. These effects are expected if prolonged exposure to a stimulus is generally associated with greater likelihood of observing new stimuli that are similar to the adaptor; in the unadapted state, there is less information about what to expect, and hence a wider range of possibilities. The expectation of both a limited stimulus range and a greater choice accuracy encourages a reduction of bound height by reducing expected difficulty of the decision. That is, the decision-making process may adjust itself to maintain the same level of expected accuracy with shorter decision times and less effort.

This bound height reduction has important implications for the interpretation of adaptation studies. For example, some adaptation experiments have not found a change in discriminability to stimuli close to the adaptor (reviewed in, Webster, 2015). However, if one does not account for response time, then it is possible that an increase of sensitivity will be masked by a simultaneous reduction of decision bounds. Indeed, we observe a change of sensitivity in our own experiment. For an example, see the sensitivity functions in Figure 6B and compare the left end of the thick red line with the dashed black line. The shift of the sensitivity function in the happy-adapted condition boosts discriminability of the happy stimuli that participants were less sensitive to in the unadapted condition. A similar conclusion holds for the sad-adapted condition (the right end of the thick blue line).

In addition to the overall reduction of decision bound, our results revealed an asymmetry of the two bounds. Participants tended to accumulate less evidence for the choice opposite to the adapting stimulus. We speculate that this asymmetry arises from two different causes—one stemming from the participant's responses following adaptation, and one from the adaptation process itself. The two explanations have different implications for the two experiments. In our first experiment, the range of test stimuli was fixed and the proportion of responses was biased: On average, participants made about twice as many judgments opposite the direction of adaptation. For a rational observer, the greater likelihood of one response over the other would lead to a reduced criterion for the more likely response, consistent with the results in Experiment 1 showing a relatively large asymmetry in decision bounds. Studies that bias the proportion of responses by altering the frequency of stimulus category also show changes in decision bounds (Hanks et al., 2011; Mulder et al., 2012; Rao et al., 2012), supporting this interpretation. If this were the *only* explanation for the change in decision bounds, we would expect the effect to be eliminated when balancing the proportion of responses, as we did in Experiment 2. As expected, this experiment did in fact show a smaller effect of bound height; however, systematic changes in bias, though smaller, were nonetheless observed even when responses were balanced, calling for an additional explanation.

The direction of changes in decision bounds is informative about the goal of adaptation. If the goal was to maintain a fixed neutral point irrespective of the local context, and if long exposure to the adapting stimulus signaled a greater likelihood of encountering similar stimuli, then it would be rational for the subject to lower his decision bound for the more likely stimulus, opposite to what we observed. However, if one purpose of adaptation is to recalibrate to the local context, such that the neutral point remains relatively close to the local mean (and therefore shifts relative to the unadapted state), then lowering the bound for the stimulus opposite adaptation would be reasonable. In other words, asymmetric decision bounds, similar to those observed in our experiments, become normative. In this view, the change in decision bound is part of the process of normalizing to the new context, complementing sensory (or representational) normalization.

If the recalibration of responses is achieved by both decision-level normalization and sensory normalization, then we would expect the change in bound height to be present immediately following adaptation, and to be present even when the proportion of responses is balanced, as in Experiment 2. This interpretation is consistent with a previous suggestion that decision-level recalibration may be a widespread (and rational) component of adaptation (Mather & Sharman, 2015). Mather and Sharman (2015) pointed out that in many domains, such as referees calling fouls in sports, sensory evidence alone can be weak, and decision factors may therefore play a large role in recalibration. In addition, the two types of effects may have different temporal dynamics: Sensory adaptation builds gradually, with longer or more repeated exposures resulting in increasingly larger effects (Rhodes, Jeffery, Clifford, & Leopold, 2007). Changes in bounds and other decision strategies, if implemented rapidly, could speed the recalibration of responses. In our experiments, the first adaptation trial in each block was long (30 s), and adaptation continued in the same direction both within and across blocks of trials, presumably leading to the accumulation of substantial sensory changes. Experiments with much briefer periods of adaptation (e.g., Raymond & Isaak, 1998) may favor a greater contribution of decision-level normalization.

A comparison of the two experiments points to an important implication for experimental design and interpretation. Selecting test probes via a method of constant stimuli spanning a fixed range is sometimes thought to be a gold standard in obtaining an unbiased measure of a psychometric function (Woodworth & Schlosberg, 1959). However, for experimental manipulations expected to shift the neutral point in a categorical decision, maintaining a fixed range of test stimuli is likely to result in a biased proportion of responses; this, in turn, is likely to cause the participant to adopt a decision-making strategy optimal for the task (for example, changing the termination bound for a decision). A wide range of experimental manipulations can shift the neutral point, such as adaptation, attention, reward, or electrical stimulation of neural subpopulations. If experimenters are interested in focusing on sensory representations, it is best to balance responses, even if it means departing from a fixed stimulus set or method of constant stimuli.

### Generalizability to adaptation of other stimulus categories

In recent years, face stimuli have been widely used for probing mechanisms of adaptation (reviewed in Webster & MacLeod, 2011). Adaptation to face properties, including emotional expression, produces large and rapid effects, similar to the dynamics observed for putatively lower level stimuli such as motion, color, orientation, and spatial frequency (Leopold, Rhodes, Muller, & Jeffery, 2005; Rhodes et al., 2007). At the same time, making categorical judgments about faces is a reasonably natural task. Thus the findings are likely to reflect the sort of adaptation processes that occur in ordinary perceptual decision-making outside the laboratory. Further experiments are needed, however, to assess the relative contribution of decisional and sensory effects underlying adaptation to other classes of stimuli or adaptation effects measured by other tasks. The novel approach we took here can easily be adapted to a wide range of other paradigms.

## Supplementary Material

Supplement 1
