# Supplementary material for: Sensory and decision-making processes underlying perceptual adaptation
Source: J Vis. 2018 Aug 22;18(8):10. doi: 10.1167/18.8.10 (PMC6108310; doi:10.1167/18.8.10)
Supplement: Supplement 1 [file jovi-18-08-05_s01.pdf]

Supplementary information

Supplementary figure 1. Related to figure 1.

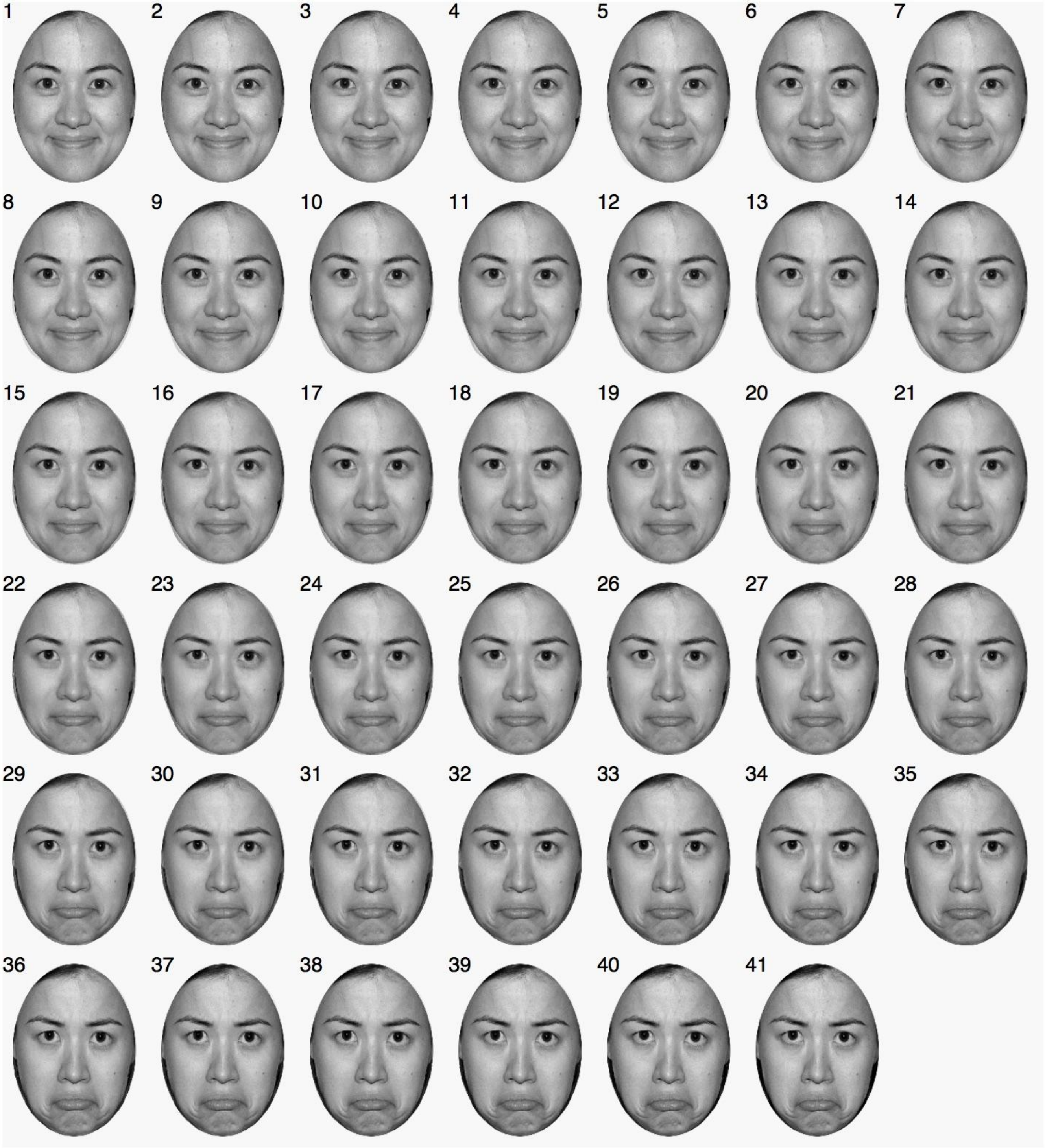

Figure S1. The 41 stimuli defining the morph line. The stimuli range from happy (upper left, stimulus strength 0) to sad (lower right, stimulus strength 1), in equal physical steps along the morph trajectory, increasing first from left to right, and then top to bottom. The two endpoints were used as adapting stimuli.

Supplementary figure 2. Related to figure 4.

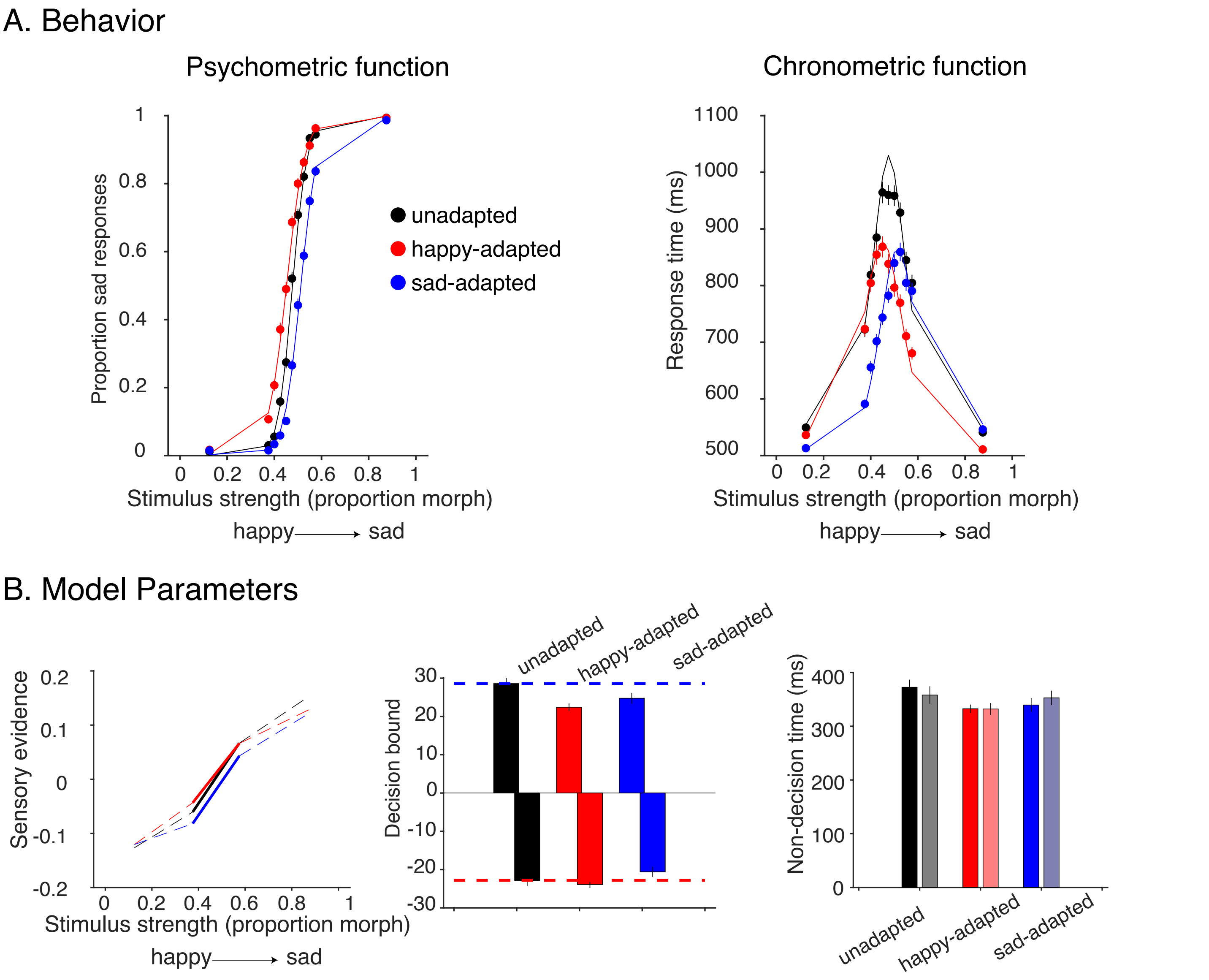

Figure S2. Model with independent non-decision times for the two choices in Experiment 1. All conventions are similar to Fig. 4, except that the model fits in panel A and model parameters in panel B are from a drift-diffusion model with different non-decision times for the two choices.

Supplementary figure 3. Related to figure 6.

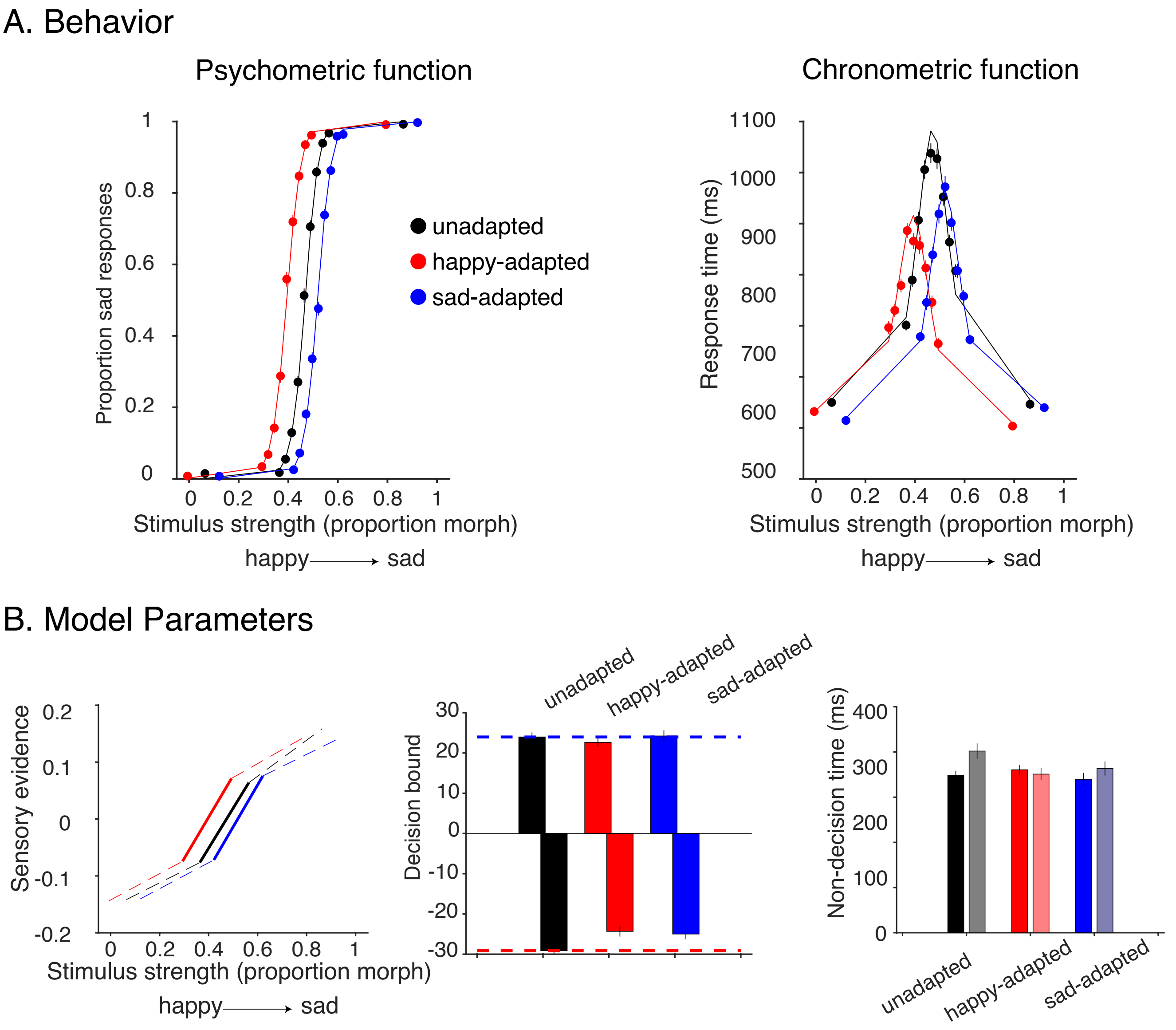

Figure S3. Model with independent non-decision times for the two choices in Experiment 2. All conventions are similar to Fig. 6, except that the model fits in panel A and model parameters in panel B are from a drift-diffusion model with different non-decision times for the two choices.

Supplementary figure 4. Related to figures 4&6.

Individual participants, Expt 2 (balanced responses)

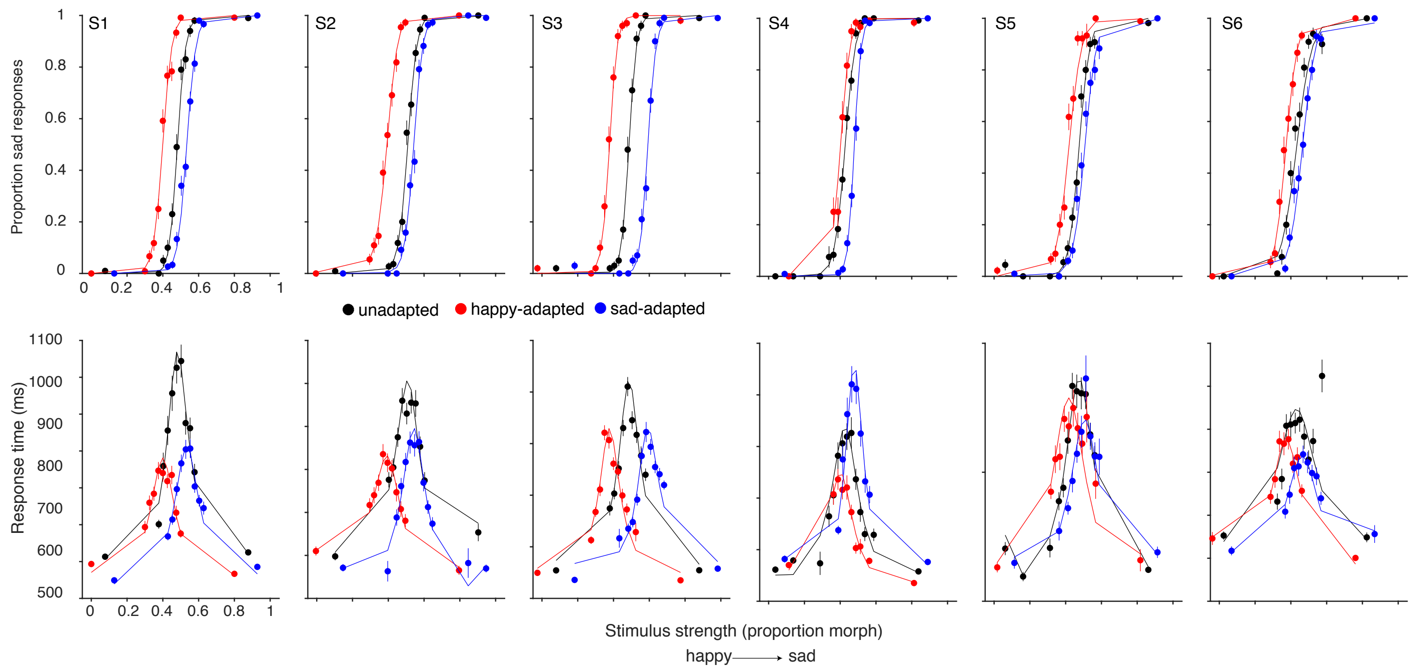

Individual participants, Expt 1 (fixed stimuli)

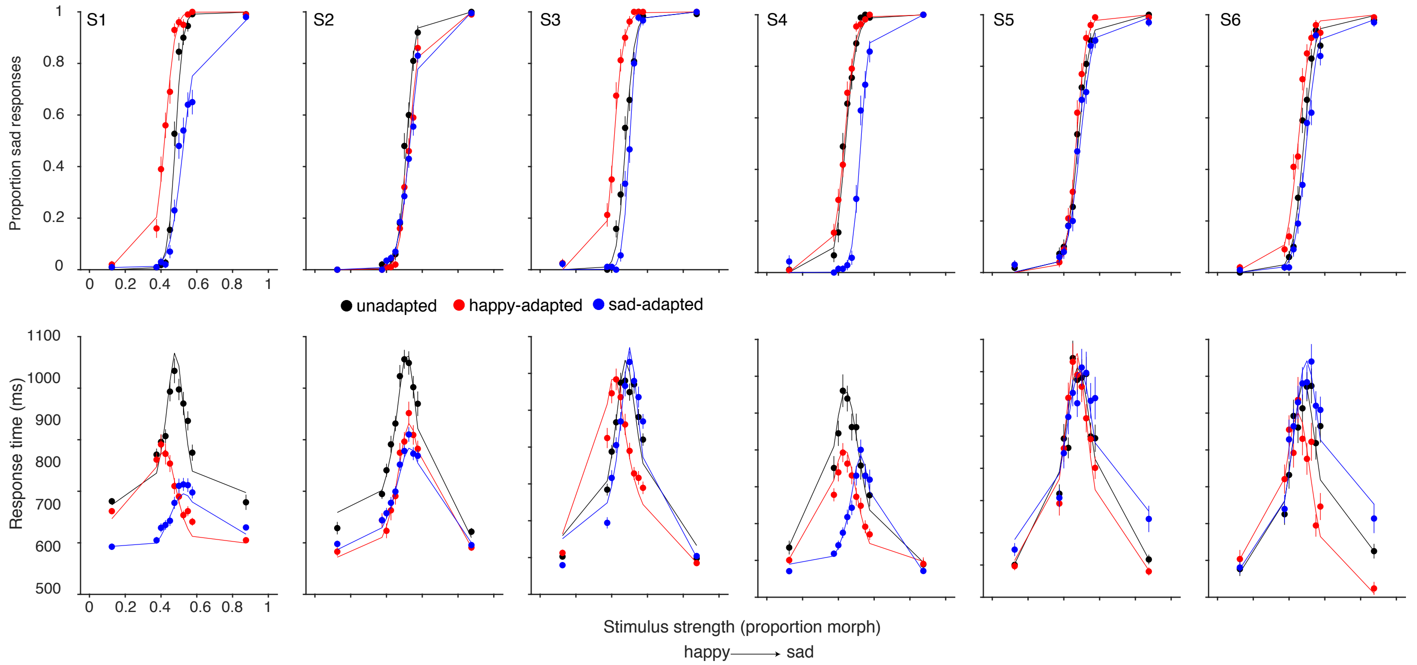

Figure S4. Individual participant data and model fits. Plots show psychometric and chronometric functions for individual participants from Experiments 1 & 2. Plotting conventions are as in Figures 4A and 6A.

Supplementary Table 1. Related to Figure 2. Psychometric functions for each participant in each experiment and adaptation condition.

		PSE			Slope		
	Participant	unadapted	happy-adapted	sad-adapted	unadapted	happy-adapted	sad-adapted
**Experiment 1, fixed stimuli**	1	0.48±0.029	0.42±0.031	0.53±0.038	46±2.6	29±2.1	22±1.6
	2	0.51±0.033	0.53±0.037	0.53±0.026	38±2.4	34±2.4	28±1.4
	3	0.48±0.027	0.41±0.036	0.50±0.033	34±1.9	34±2.8	41±2.7
	4	0.44±0.031	0.43±0.028	0.52±0.042	36±2.5	33±2.1	32±2.6
	5	0.48±0.029	0.46±0.029	0.49±0.033	26±1.6	31±1.9	22±1.5
	6	0.48±0.030	0.45±0.030	0.50±0.032	29±1.8	26±1.7	26±1.7
	Pooled	0.48±0.012	0.45±0.013	0.51±0.013	31±0.76	24±0.65	26±0.68
**Experiment 2, balanced responses**	1	0.48±0.029	0.40±0.023	0.53±0.027	37±2.2	35±2.0	36±1.8
	2	0.50±0.030	0.40±0.023	0.55±0.031	37±2.2	32±1.9	36±2.0
	3	0.48±0.030	0.37±0.023	0.58±0.036	40±2.5	33±2.1	33±2.0
	4	0.44±0.026	0.41±0.040	0.49±0.030	37±2.2	33±3.2	48±2.9
	5	0.46±0.029	0.42±0.029	0.51±0.033	24±1.5	27±1.8	26±1.7
	6	0.42±0.026	0.36±0.024	0.47±0.030	27±1.7	29±1.9	26±1.7
	Pooled	0.46±0.012	0.39±0.010	0.52±0.012	33±0.81	32±0.84	33±0.79

Table S1. Point of subjective equality (PSE) and slope for each observer ± standard error from bootstrapping.
